# Triple helical DNA in a duplex context and base pair opening

**DOI:** 10.1093/nar/gku848

**Published:** 2014-09-16

**Authors:** Mauricio Esguerra, Lennart Nilsson, Alessandra Villa

**Affiliations:** Department of Biosciences and Nutrition, Karolinska Institutet, Hälsovägen 7, SE-141 83 Huddinge, Sweden

## Abstract

It is fundamental to explore in atomic detail the behavior of DNA triple helices as a means to understand the role they might play *in vivo* and to better engineer their use in genetic technologies, such as antigene therapy. To this aim we have performed atomistic simulations of a purine-rich antiparallel triple helix stretch of 10 base triplets flanked by canonical Watson–Crick double helices. At the same time we have explored the thermodynamic behavior of a flipping Watson–Crick base pair in the context of the triple and double helix. The third strand can be accommodated in a B-like duplex conformation. Upon binding, the double helix changes shape, and becomes more rigid. The triple-helical region increases its major groove width mainly by oversliding in the negative direction. The resulting conformations are somewhere between the A and B conformations with base pairs remaining almost perpendicular to the helical axis. The neighboring duplex regions maintain a B DNA conformation. Base pair opening in the duplex regions is more probable than in the triplex and binding of the Hoogsteen strand does not influence base pair breathing in the neighboring duplex region.

## INTRODUCTION

Abnormal gene expression often leads to disease. Silencing the expression of specific genes is starting (1) to be used to treat human disease and promises to have a tremendous effect on the treatment of critical diseases, such as cancer. Gene expression can be regulated by targeting genomic DNA with ligands, for example, proteins. Among DNA binders triplex-forming oligonucleotides (TFOs) are major groove ligands which target specific DNA sequences by forming DNA triplexes (2–4). This ability has considerable biotechnological and therapeutic potential (5,6) and has been extensively studied for use in applications, such as transcription modulation and site-directed recombination as well as mutagen delivery (7,8).

A DNA triplex is a helical structure composed of three strands in which a single DNA strand binds to the major-groove of a Watson–Crick duplex. The third strand bases hydrogen-bond to the duplex purine strand, forming Hoogsteen or reverse Hoogsteen pairs. Triplex formation can come in different ways: intramolecular or intermolecular, with purine or pyrimidine motifs, in parallel or anti-parallel orientations (9).

The TFO approach, as anti-gene tool, has some sequence restrictions, since TFOs are only able to target stretches of homo-purine·homo-pyrimidine bases. An alternative way to target DNA and overcome sequence restriction is the use of clamp constructs. These molecules have the capacity to bind to double-stranded DNA and strand invade it (10). Recently, Moreno *et al.* (11) developed a clamp oligonucleotide molecule, formed by a triplex-forming (Hoogsteen-binding) arm and a strand invading Watson–Crick arm linked together. The mechanism used by clamp molecules to target helical DNA is most likely the following (12): first, the TFO arm binds to the major groove of helical DNA, then the Watson–Crick arm strand invades double helical DNA.

**Figure 1.**
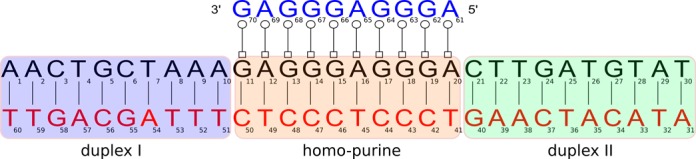
Secondary structure representation of the simulated system including the anti-parallel homo-purine region using Leontis–Westhof annotation (13). Note that the hollow square and circle represent a base pair in the trans geometry, that is, a reverse Hoogsteen base pair. As a convention we designate the leading strand as the one colored black, the complementary strand red and the Hoogsteen strand blue. The highlighted regions in magenta (first duplex), orange (homo-purine) and green (second duplex) are presented in the structural analyses.

Knowing how the conformations of double-stranded DNAs change upon binding of TFOs, and how the presence of a third strand influences base flipping (first step for strand invasion) in different regions of DNA duplexes contributes to understand how clamp compounds work and might help on designing strategies to improve both binding and strand invasion efficiency. To achieve this we have used molecular simulations techniques to investigate, at atomistic level, a 10 base single-stranded DNA (TFO) targeting a 30-mer DNA double strand and forming an anti-parallel triplex with the purine-motif (secondary structure using Leontis–Westhof annotation (13) in Figure 1). We have selected a sequence with high guanine content to guarantee triplex stability (14,15). To mimic two possible extreme scenarios that may happen in cellular environments, we have simulated DNA helices as an isolated and continuous molecule. To assess strand invasion efficiency, we have investigated the flipping of base pairs in the ligand target site, and neighboring the target site. Umbrella sampling techniques were used to generate free-energy profiles for base flipping both in the major and minor grooves. We focus on the thymine:adenine base pair, since this base pair has higher flipping probability, and is most probably a preferred site for strand invasion.

Structural information on short triplexes with purine motif is available from standard techniques, such as nuclear magnetic resonance (NMR) spectroscopy and X-ray crystallography, and molecular simulations (16–19). In the purine motif, G-G·C and A-A·T triplets are formed in a reverse Hoogsteen conformation, (‘−’ refers to Reverse Hoogsteen and ‘·’ to Watson–Crick). The resulting triple-helix has the TFO (or Hoogsteen) strand oriented in opposite direction (anti-parallel orientation) to the 5′ to 3′ sense of the duplex homo-purine strand. In contrast to the pyrimidine motif, the purine motif is pH-independent and has been used more often for *in vitro* inhibition of transcription (20). Triplexes with the purine motif have been observed *in vivo* and linked to human disease (21), e.g. Friedreich's ataxia, a neuro-degenerative disease caused by a large expansion of the tri-purine repeat (22).

First, we discuss in detail local and global structural features of DNA helices in the presence or absence of TFO to address the question of whether an anti-parallel third strand will easily accommodate in a B DNA helical conformation, or if binding will be facilitated in another type of conformation. We have performed a careful analysis of DNA geometry at the base pair step level using the rigid-body approach as is nowadays customary in the field (23,24). As pointed out by Lu and Olson (23) due to irregularities found in ‘real’ (long and dynamic) DNA structures, it is necessary to perform careful structural analyses to prevent imprecise conformational assignments. In the second part, we focus on the influence of the third strand on base pair opening. In particular, we discuss the equilibrium shift between opening and closing state of a base pair upon binding and how the presence of TFO may affect strand invasion efficiency.

## MATERIALS AND METHODS

### Molecular dynamics (MD) simulations

A 30-mer DNA duplex alone and in complex with a 10-mer third DNA strand was simulated in water solution with and without a helical Twist constraint. To impose a helical Twist constraint the DNA duplex has been treated as a continuous polymer. Meaning that the DNA helix is extended from one end of the simulation volume to the opposite end, where it continues through normal bonding connectivity (25). We label the system with helical Twist constraint, *continuous* helix, and the one without, *isolated* helix.

All MD simulations were performed using the GROMACS suite of programs (version 4.5) (26,27). The CHARMM27 force field (28,29) was employed to describe the DNA structures and sodium ions. The DNA systems were placed in a cubic box of 12 nm. A rectangular box of 9 nm height was used for the *continuous* DNA systems. The boxes were subsequently filled with TIP3P water molecules (30). To neutralize the system, sodium ions were placed randomly in the simulation box.

The particle mesh Ewald method (31) was employed to treat Coulomb interactions using a switching distance of 1.0 nm and a grid of 0.12 nm. Lennard–Jones interactions were switched off between 1.0 and 1.2 nm. Constant pressure *p* and temperature *T* were maintained by weakly coupling the system to an external bath at 1 bar and 300 K, using the Berendsen barostat (32) and velocity-rescaling thermostat (33), respectively. The system was coupled to the temperature bath with a coupling time of 0.1 ps. The pressure coupling time was 1.0 ps and the isothermal compressibility 4.5· 10^−5^ bar^−1^. A semi-isotropic pressure coupling was applied in the case of the *continuous* DNA system (that is isotropic in the *x* and *y* directions, but not in the *z* direction).

Bond distances and angles of water were constrained using the SETTLE algorithm (34). Other bond distances were constrained using the LINCS algorithm (35). A leap-frog integrator with an integration time step of 2 fs was used.

The starting structures of 30-mer 5′AACTGCTAAA-GAGGGAGGGA-CTTGATGTAT 3′ and 10-mer 5′AGGGAGGGAG 3′ DNA were generated in the B form as a double strand and single strand, respectively, using the software package 3DNA (23). To generate the starting structure of DNA triplex, 10 ns MD simulations were performed using distance restraints between the hydrogen-bond donor and acceptor atoms of the Watson–Crick side of the 30-mer purine and the reverse-Hoogsteen side of the third strand. After 10 ns equilibration, 50 ns of MD simulation were performed for each system.

### Structural analysis

To avoid possible misclassification of DNA conformations as mentioned in the introduction, we analyze DNA double helical steps in our MD trajectories using the 3DNA (23,36) software and its set of *ruby* scripts called x3dna_ensemble. Graphs and statistical analysis were produced using home-brewed scripts written in **R** (37).

Base pair parameters for Watson–Crick steps and Hoogsteen steps were determined for each base pair and time-averaged. For convenience the DNA helix was divided in three regions: first duplex, homo-purine and second duplex. Average step parameters for each region were obtained by averaging on the base pair time-averaged values. The standard error of the mean was calculated using block averaging (38).

For fast and detailed interpretation of the conformational space of base pair steps we explore the local helical parameters Inclination versus *x*-displacement, the base-step parameters Roll versus Slide (39) and the local axis interstrand phosphorus to phosphorus vector projections $z$_*p*_(*h*) versus $z$_*p*_ for every step in the simulated DNAs. Slide (*Dx*) can discriminate between B DNA and A DNA conformations since their mean values are widely spaced apart, whereas Roll (*ρ*) can discriminate A DNA and B DNA apart from TA-DNA but not between them (40). It has been show that the phosphorus to phosphorus vector projection on the $z$-axis of the local helical axis reference frame $z$_*p*_(*h*), discriminates effectively between B DNA and TA-DNA conformations (41), and that the corresponding projection on the middle-step reference frame $z$_*p*_ differentiates between A DNA and B DNA conformations (42).

To analyze global features major and minor groove widths are computed as inter-strand phosphorus to phosphorus distances (43). These values can be correlated with local features described by the base pair step parameters. Additionally, we present endocyclic as well as exocyclic base pair step overlaps which correlate with DNA base-stacking geometries and interactions (23,39).

To quantify the effect of the third strand on helical structure, angles between the centers of mass of Watson–Crick base pairs are calculated. In particular, we have computed the angles between the middle base pair (position 15 in the 30-mer DNA) and equidistant neighboring base pairs.

To compare the conformational fluctuations of helical structure in the presence and absence of TFO we have performed a principal component analysis (PCA) (44–46) of the double-strand DNA merged trajectories. All the atoms are considered. Before performing the analysis each coordinate set in the trajectory is translated and rotated to give the best fit to the middle helical frame (between base pairs 11 and 20) of the duplex reference structure. The first four (of 5715 in total) eigenvectors describe 86 % of total fluctuations.

### Potential of mean force (PMF)

To model base flipping the PMF was computed using umbrella sampling with a harmonic potential bias $w$_*i*_ = *k*(*x* − *x*_*i*_)^2^/2 along a reaction coordinate *x*, defined as a pseudo-dihedral angle between the center of mass of the flipping base, of the corresponding sugar, of the first neighboring sugar and of first neighboring base pair. This reaction coordinate has widely been used to study base flipping in DNA and RNA (47–49) (for a detailed definition see (47)). The PMF calculations were performed using GROMACS suite of programs (version 4.5) using dihedral restraints, that work on the center of mass through virtual interaction sites.

Base flipping PMFs were obtained by performing 72 independent simulation windows varying the reference pseudo-dihedral angle *x*_*i*_, from −180° to 180° in 5° intervals. Initial conformations for every window were generated by running 5 ps each along the reaction coordinate with *k* = 20 000 kcal/(mol rad^2^). In the production phase, each window was run for 5 ns (of which the last 4 ns were used for analysis) with *k* = 2000 kcal/(mol rad^2^). Dihedral restraint, that works on the center of mass through virtual interaction sites, was used. The values for the pseudo-dihedral angle were recorded every step. The PMF curves were generated from the resulting distance distributions using the Weighted Histogram Analysis Method (50,51) with a tolerance of 10^−6^, 720 bins and enforcing periodicity of the reaction coordinate. Error bars were obtained by dividing data collection in each window to four parts and computing their standard deviation.

Free energy changes in base pair opening were calculated by integration of the probability distribution function (obtained from the PMF curve) over the closed and open states for A and T flipping. To define open and closed states for the base pair, we calculated the solvent accessible surface area of the H3 atom in pyrimidine and N1 in purine. The atomic solvent accessible surface area was computed numerically (52) setting the solvent probe to a radius of 0.14 nm for carbon, 0.13 nm for oxygen and phosphorus and 0.10 nm for hydrogen. A closed base pair is defined as having an atom solvent accessible surface area of less than 0.001 nm^2^.

The energy barriers for base flipping were defined by the difference between the minimum and the maximum values of the PMF curve.

## RESULTS AND DISCUSSION

**Figure 2.**
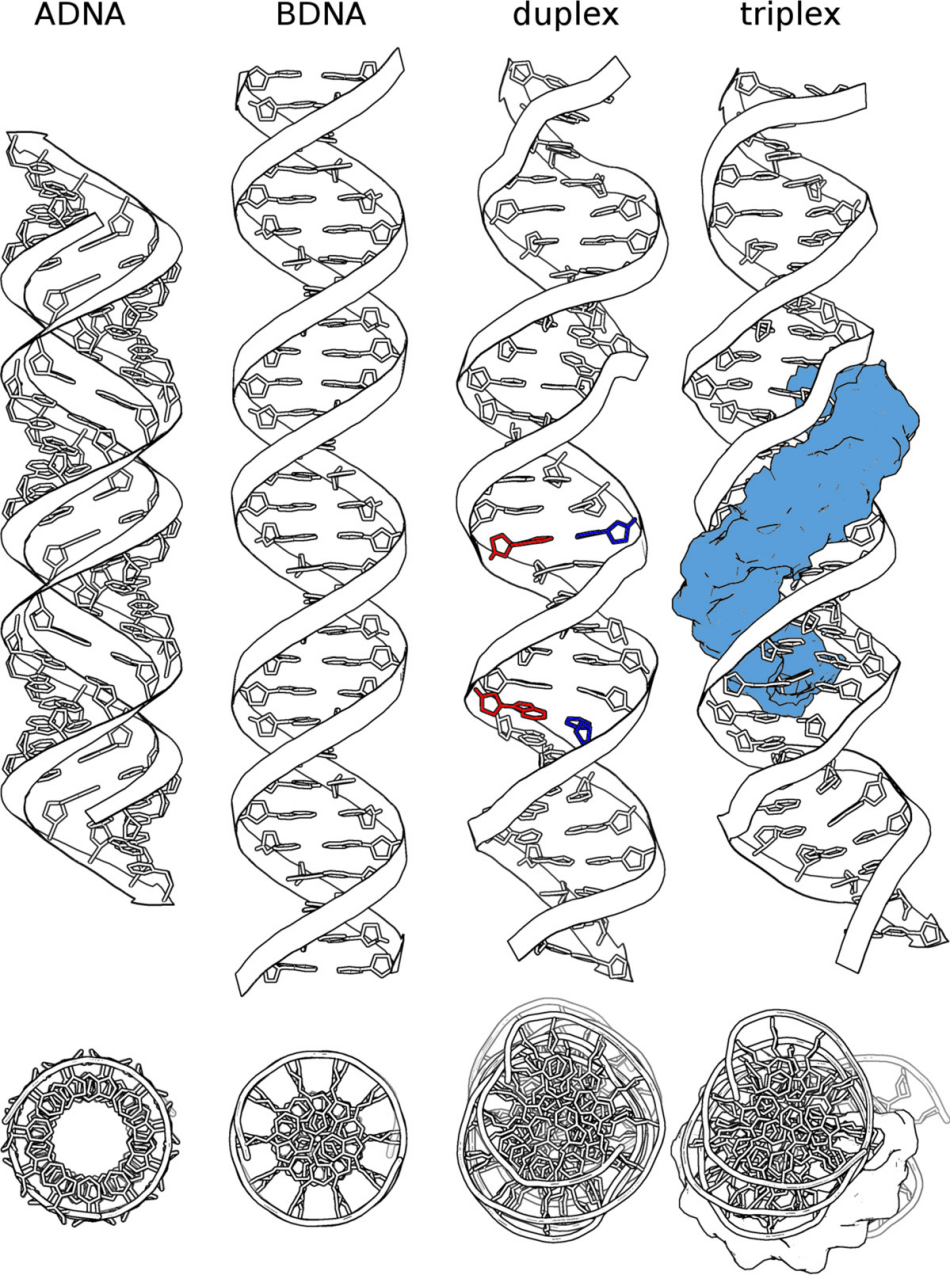
Standard fiber-model conformations of DNA compared to the average structure of the simulated *isolated* duplex and triplex. The middle helical frame between base pairs 11 and 19 has been used as reference-frame for alignment of all structures. Adenine is colored red and Thymine blue in the base pairs belonging to the flipping potential study (A_16_·T_45_ and T_22_·A_39_). The Hoogsteen strand is displayed as a blue surface and it can be noted that the triplex ‘accepting’ region in the duplex seems to be somewhat ‘preformed’ into the shape the Hoogsteen strand will ultimately occupy. The arrowheads in the ribbons correspond to 3′ ends.

DNA helical systems were simulated in explicit solvent in *isolated* and *continuous* fashion, with and without TFO in the binding site. Simulating DNA helices as continuous molecules prevents DNA from non-intrinsic twisting and removes end-effects. The last 50 ns have been used for structural analysis. Figure 2 shows the average structure from the simulations for the *isolated* helical system in presence or absence of the third DNA strand. In the duplex systems, the bases of the leading strand form Watson–Crick base pairs with the opposite strand. An infrequent spontaneous opening of a base pair at the ends is observed in the *isolated* helix. In the triplex systems, the third strand is bound to the targeted region in an anti-parallel fashion, forming reverse Hoogsteen base pairs with the leading strand. Loss of hydrogen bonds between Watson–Crick and Hoogsteen strands is observed for the end residues of the TFO (A69 and G70). This may be due to the length of the TFO. Indeed, this effect was not reported in simulations (performed with the same force field) on parallel triplexes with 17-mer TFO binding a 32-mer duplex (53).

### Local structural analysis

For convenience we refer to three regions in the simulated system; first duplex, homo-purine and second duplex (Figure 1). Base pair parameters for the Watson–Crick steps were determined (Supplementary Tables S1 to S4). Twist and Roll values at the homo-purine region (TFO binding site) are slightly affected by the presence of the third strand. Twist average value decreases from 35.5° to 33.1° for *isolated* helices, Roll value increases from 4.7° to 5.6°, while Rise is constant at 3.30 Å. A similar trend is observed for the *continuous* helices: Twist decreases from 36.6° to 34.5°, Roll increases from 4.3° to 5.6° and Rise is constant at 3.30 Å. The observed differences are slightly larger than the corresponding standard error of the mean (0.2–1.0° for Twist, 0.2–0.7° for Roll and 0.01–0.05 Å for Rise.) This indicates that the presence of the TFO moves step parameters values from B DNA toward an A DNA conformation. Reference values (54) for Twist, Roll and Rise in B DNA and A DNA are 36.0 and 31.1 degrees, 0.6 and 8.0 degrees, 3.32 and 3.31 Å, respectively. Similar trends for step parameters were also reported in previous simulation studies on triple helical systems (19,55).

Base pair step parameters for reverse Hoogsteen steps (Supplementary Tables S5 and S6) were also determined. Average values for Twist and Rise are 32.8° and 3.3 Å, respectively (32.6° and 3.3 Å in *continuous* systems), indicating a clear deviation of the third strand from B DNA conformation.

#### Classification of double helical steps

**Figure 3.**
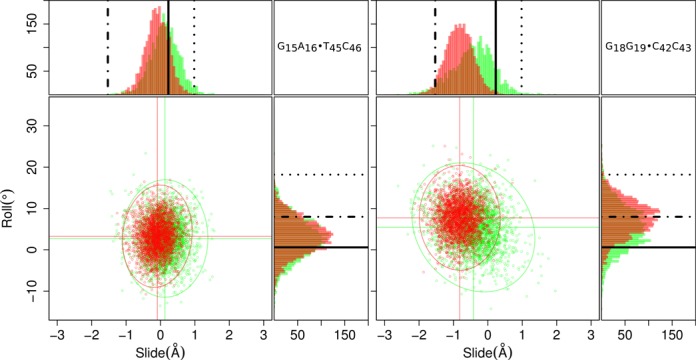
An example of the base pair step parameter distributions explored in this study highlights the remarkable resistance to deformation on hybridization of the Hoogsteen strand of GA·TC base pair steps. These steps (left-hand side scatterplot) remain close to mean values of Roll (*ρ*) and Slide (*D*_*x*_) typical of the B DNA conformation (23) as indicated by the black solid line in the histograms, whereas the GG·CC steps (right-hand side scatterplot) deform to values far away from the B DNA conformation via negative oversliding. The ellipses are projections of the harmonic equi-potential surface derived from the covariance matrix of Roll–Slide values and correspond to scores of 4.5 *κ_β_T* or 3*σ* away from the mean (Olson *et al.* (56)). Histograms and points are colored green for the *isolated* duplex, and red for the *isolated* triplex. Standard conformational values are indicated by a dash-dot line for A DNA, solid for B DNA and dotted for TA-DNA. A hundred bins are populated between the upper and lower bounds of Roll and Slide in the histograms.

To explore the full extent of the behavior of every base pair step in the simulations, scatterplots and histograms are plotted for all parameters (Supplementary Figures S1–S9). As example, two characteristic cases are highlighted in Figure 3. A bird's-eye view inspection of the graphs shows Gaussian and unimodal distributions with no significant differences in *continuous* versus *isolated* cases. The only case which defies the rule is the AT·TA step at the end of the second duplex region. This makes sense as it is located at the end of a helical region and it is well known that such flanks are more floppy. On the other hand, this is a purine-pyrimidine (RY) step which intrinsically behaves as a rigid step (56–58) and perhaps should be closer in behavior to the AA·TT step at the beginning of the first duplex, also known to be relatively rigid.

The base pair steps linking the duplex regions and homo-purine region (AG·CT and AC·GT steps) undergo a change in environment upon TFO binding, from pure Watson–Crick base paring to a canonical and non-canonical base pairing (when the TFO is bound to the homo-purine region), nonetheless no significant differences are observed in the distributions of base pair step parameters.

We see in the plot of Roll versus Slide for the AG·CT linker step (Supplementary Figure S4) that the distribution corresponding to the *continuous* duplex has Slide values closer to a mean value of zero instead of the negative mean value seen in the other contexts.

**Figure 4.**
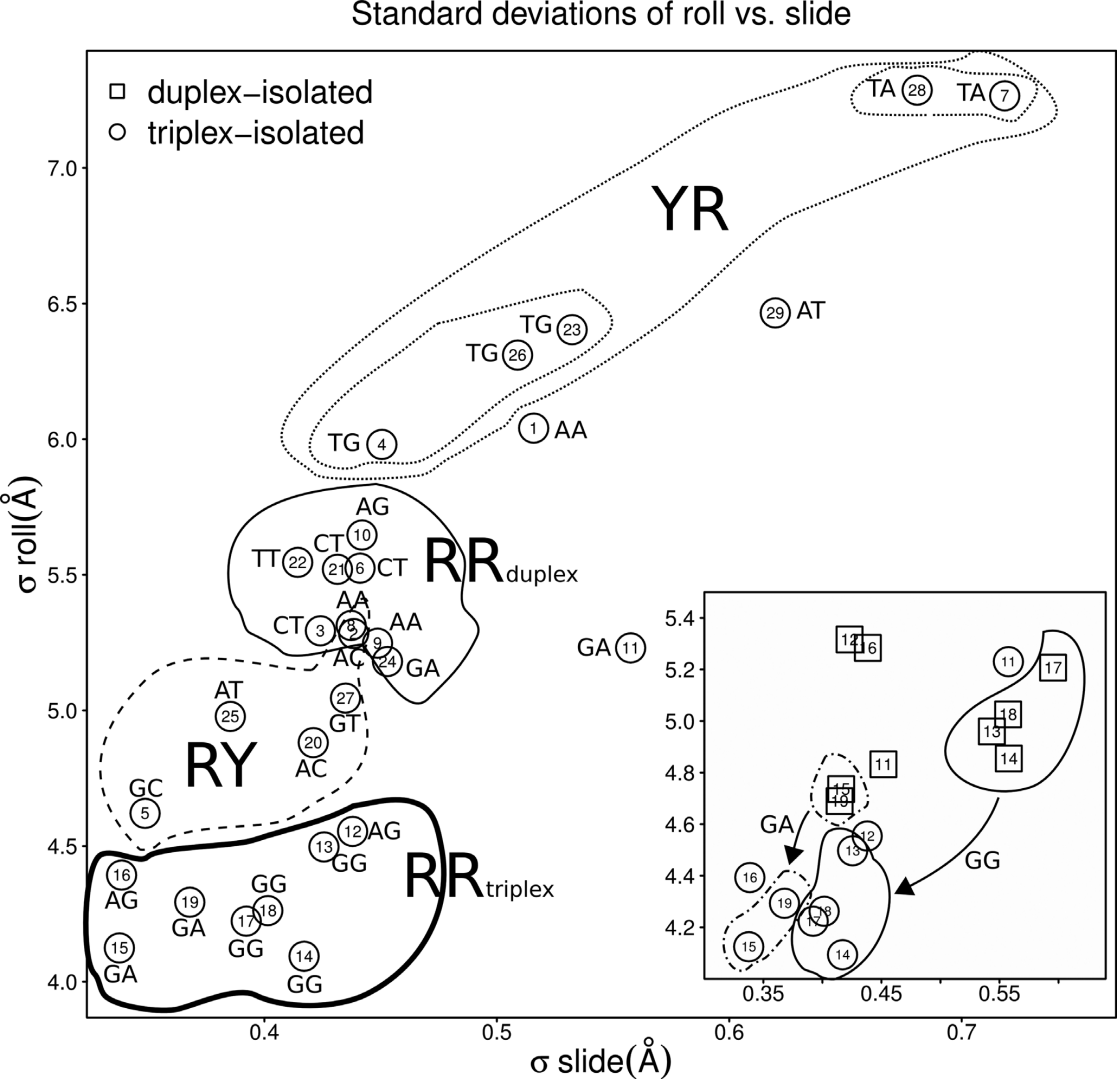
Variabilities in Roll and Slide given by the standard deviation of the rotational and translational parameters along the long axis of the base pair steps. Nine out of 10 possible unique base pair step parameters are included in the Watson–Crick paired regions of the simulated systems (only CG is missing) and follow well known trends on deformability. Pyrimidine-purine (YR) steps are most deformable, purine-purine (RR) are rigid and purine-pyrimidine (RY) are most rigid as clearly gathered from the plot. The plot displays nicely how the RR steps in the context of the homo-purine region (delimited by a thick solid black line and numbered 12–19) become more rigid and have smaller variabilities than their triplex-context free counterparts. In the inset we see this loss of deformability for the RR steps GG and GA when the variability of duplex-only (squares) and flanked-triplex systems (circles) are compared.

In general, the trends in the duplex regions are as expected (56,59), that is, pyrimidine-purine (YR) steps are the most flexible judging from the spread of the data. This can be easily appreciated at YR steps TG·CA and TA·TA in the first and second duplex regions (Figure 4).

As shown by Slide values the sampled space of duplex-only regions stays close to the standard B DNA conformation (highlighted by a vertical solid line in Supplementary Figures S4–S6). No conformations similar to the TA-DNA type are observed as clearly indicated by Roll. When looking at the local helical parameters *x*-displacement (*dx*) and inclination (*η*) (Supplementary Figures S1–S3) one sees a parallel story to the one told by the base pair step parameters Slide and Roll, that is, in the duplex regions *dx* values are closer to B DNA standard values, and there are no conformations close to the mean value of *η* for TA-DNA. In the homo-purine region, the base pair step parameter Slide shifts slightly toward negative values, indicating a sort of pre-condition of the region to accommodate a third strand (see below).

Not surprisingly due to the influence of the Hoogsteen strand in the homo-purine region significant differences are seen in Slide and *x*-displacement between duplex and triplex cases and a barely notable difference between *continuous* and *isolated* cases for the triplex system at the second, third and fourth steps. No significant differences are seen in Roll or Inclination. Slide shifts its mean value in the negative direction toward the values common for A DNA and the same trend is observed for *x*-displacement. Surprisingly, the three GA·TC steps in the homo-purine region resist oversliding (Figure 3). In general, the trends for purine-purine (RR) steps agree with those recently reviewed by Balasubramanian and Olson (58), that is, AG·CT steps are positively correlated, GG·CC steps are negatively correlated and GA·TC have a smaller positive correlation. The shape of Slide and *x*-displacement distributions for steps in the triplex region remain the same but are overslid or overdisplaced in the negative direction.

The $z$_*p*_(*h*) versus $z$_*p*_ scatterplots display no major differences in *continuous* versus *isolated* cases, and duplex versus triplex (Supplementary Figures S7–S9). There is a very small difference, nevertheless, at the GG·CC steps in the homo-purine region where $z$_*p*_(*h*) for triplex cases are slightly larger than their duplex counterparts. This is clearly due to a major groove widening accompanying oversliding. In general, the values of the interstrand phosphate to phosphate vector projection metrics remain in the B DNA and AB conformational regions with some few cases sampling TA-DNA, and in rare cases the A DNA areas.

It is important to note here then that the oversliding ‘deformation’ due to the presence of the Hoogsteen strand does not lead the Watson–Crick double helix to an A DNA conformation nor a B DNA one, but rather to a B-like conformation in the area of transition between A and B conformations whose base pair planes are slightly slanted but close to being parallel to the helical axis and with large *x*-displacement (Figure 2).

A qualitative indication of base pair step sequence preferences can be inferred from the analysis of the average endocyclic and exocyclic overlaps (Supplementary Figures S10 and S11). Endocyclic overlaps give us an idea of ‘mainly’ geometrical preferences acquired by base pair steps via rolling (*ρ*), sliding (*Dx*) and twisting (*ω*) (39), whereas exocyclic overlaps might additionally correlate to electrostatic interactions of the highly polar exocyclic groups, e.g. as seen in base pair electrostatic potential plots (60). Average overlaps (Supplementary Figure S10) correlate with well known trends for the deformability of DNA's base pair steps, that is, YR steps are the most deformable and therefore have the smallest overlaps, RR steps are more rigid and have larger overlaps and RY steps are the most rigid (56) and have relatively large overlaps. Looking into both duplex regions we can say that the main trend proposed by Calladine rules (59) is followed, that is, to avoid purine-purine atomic clashes from opposite strands the YR steps deform more easily into the major groove while the RY steps are more rigid and deform toward the minor groove. For the triplex area, considering that all such steps are of the RR type and of intermediate stiffness (61,62), we see that their values are in the same range as those for RY. All throughout it can be seen that the trends followed by the overlap of base pair steps remain fairly the same in the duplex as well as in the homo-purine region when the Hoogsteen strand is present and in its absence as can be judged by the spread of the overlap (Supplementary Figures S10 and S11).

A richer picture to describe the flexibility of base pair steps in the simulated systems emerges when one plots a simple diagram of the spread of the data in the Roll and Slide dimensions (Figure 4). It can clearly be seen that the variabilities in Roll and Slide are positively correlated, that is, an increase in the variability of Slide, results in an increase in Roll variability for YR steps when compared to RY steps and furthermore they discriminate within unique base pair steps corresponding to the same ‘family’, that is, looking at the pyrimidine-purine steps in the duplex regions we see that TA steps are more flexible than TG steps. RR steps in the context of the homo-purine region become more rigid upon binding and have smaller variabilities than their triplex-context free counterparts.

El Hassan and Calladine (39) showed from the analysis of 60 DNA structures that bimodality may occur in GG, GC or CG steps due to unfavorable stacking interactions. Interestingly, we see no bimodality as suggested by El Hassan and Calladine. We only have one GC step in the first duplex region, and four GG steps in the homo-purine region, but neither on the duplex-alone, nor in the triplex cases, do we observe appreciable bimodality.

### Global analysis

**Figure 5.**
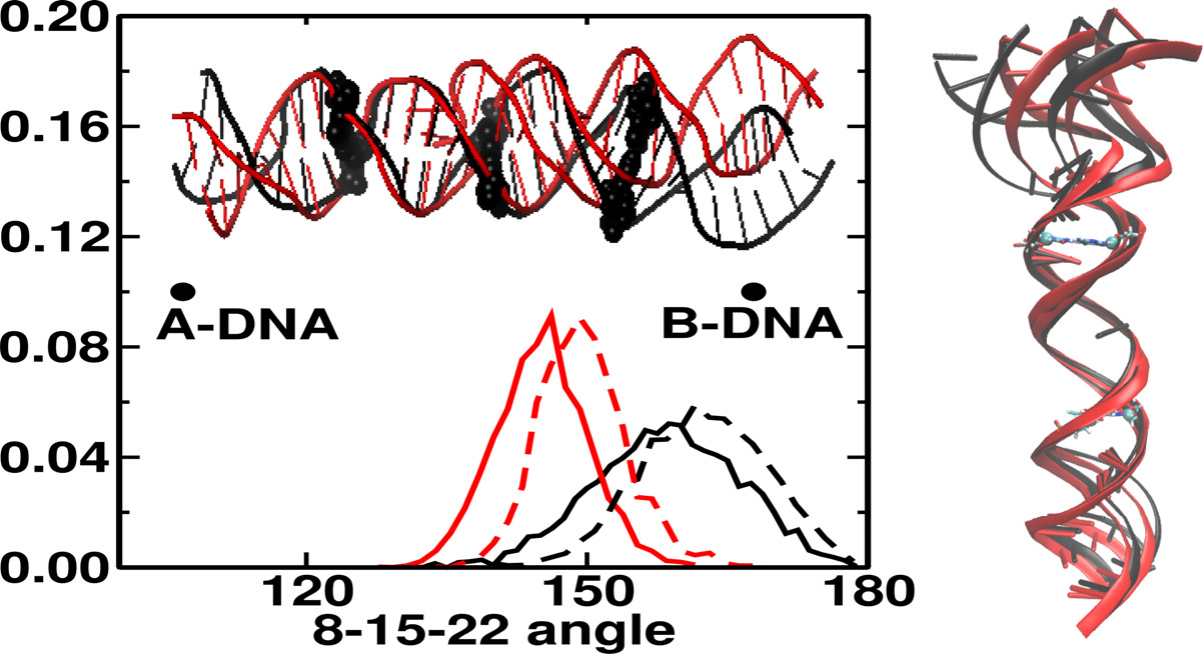
Global structure analysis. Left side, distribution of the angle (°) between the center of mass of base pairs in position 8, 15 and 22. Values for *isolated* (continuous line) and *continuous* (dashed line) DNA duplex and triplex are in black and red, respectively. Values for a generic A DNA and B DNA structure are also reported as reference. Insert snapshot at 50 ns of DNA double (black) and triplex (red) in cartoons representation. The base pairs in position 8, 15 and 22 in VDW drawing style. Right side: two extreme projections of the first eigenvector along trajectory of the duplex (black) and triplex (red). Base pair in position 11 and 20 in licorice and corresponding C1′ in VDW drawing style.

To quantify the effect of the third strand on global structure, we have calculated helical angles with respect to the middle base pair G15·C46 (Figure 5, left panel). Differences between duplex and triplex are observed when the angles involve base pairs located between position 7 and 23. In particular, the binding of the TFO lowers the angle values and narrows the distribution. Values for the duplex are slightly smaller than for B DNA conformation, while triplex values move toward A DNA conformation. Larger differences ∼15° are observed for angles involving base pairs neighboring the TFO binding site (i.e. angle between positions 8-15-22 or 9-15-21), for both *isolated* and *continuous* DNA.

This indicates a change in the curvature of global helical structure to allocate the third strand in the major groove (Figures 2 and 5), but also a rigidity of the helical structure upon TFO binding, as shown by the narrowing of the distribution (Figure 5).

To further investigate the TFO effect on the fluctuations of the DNA duplex, we have performed a PCA of the merged trajectory of the duplex DNA in absence or presence of TFO. Differences are mainly observed along the first eigenvector (32% of the total fluctuations). The presence of the ligand shifts and narrows the distribution along the first eigenvector (Supplementary Figure S12) while no significant difference is observed in the distributions along the other eigenvectors. The motion along the first eigenvector is illustrated in Figure 5 (right side) and it can be described as a sort of bending of duplex I in the C1′—C1′ direction of base pair 11 (first base pair of the homo-purine region) and bending of duplex II in the C1′—C1′ direction of base pair 20 (last base pair of homo-purine region). The change in helical global structure upon binding of the third strand is probably determined by the local change observed in the TFO-target region, where differences in Slide are observed between duplex and triplex. The reduction in fluctuation of the helical structure reflects the increase in rigidity observed for Roll and Slide of purine-purine steps in the triplex regions (Figure 4).

**Figure 6.**
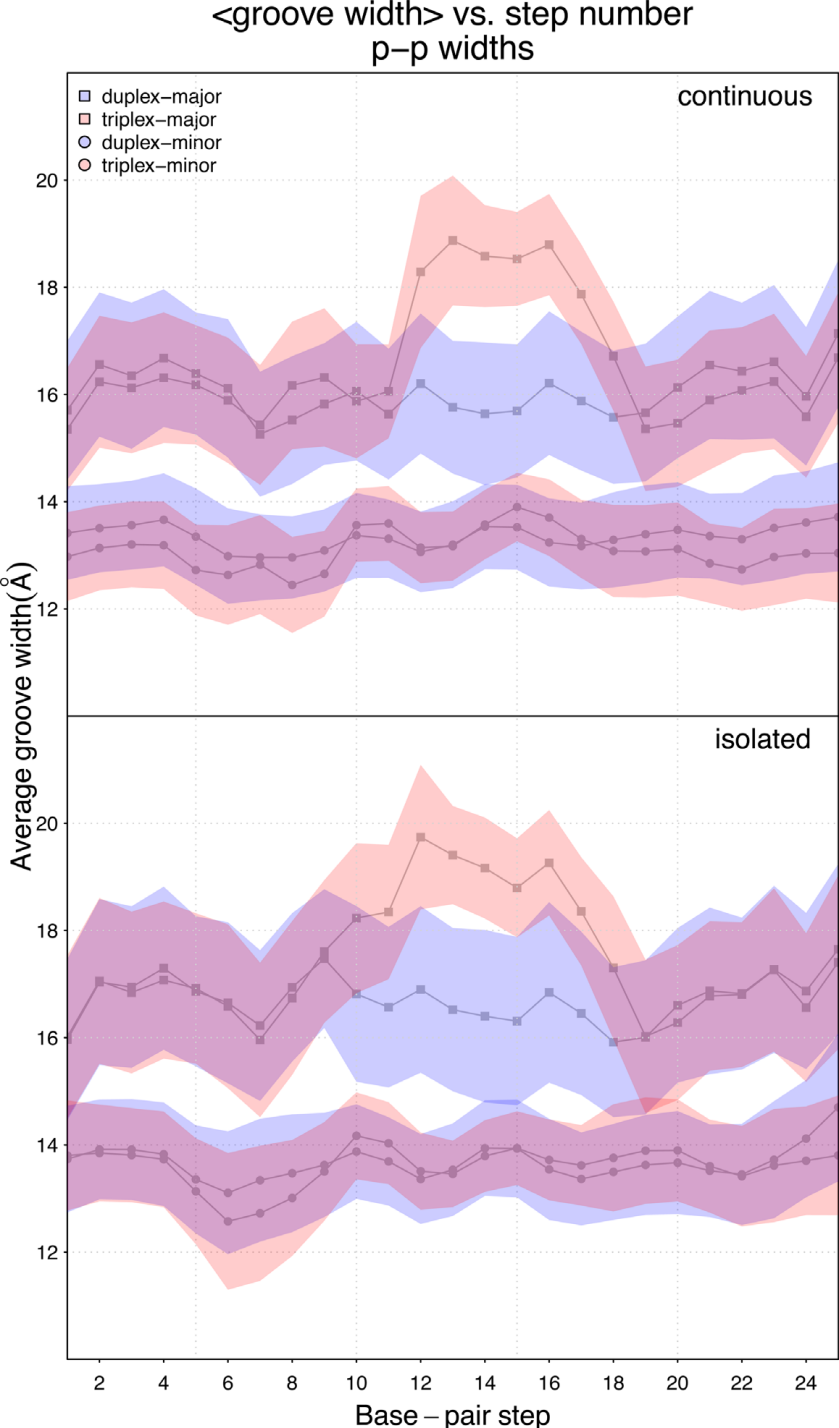
Minor and major groove widths in the duplex regions do not vary when comparing the duplex alone and triplex systems. The major groove in the homo-purine region increases by about 2 Å when the Hoogsteen strand is present. The colored areas correspond to one standard deviation away from the mean groove values.

Global features are also explored via major and minor groove-widths (Figure 6). We see that in the duplex regions within one standard deviation from the mean groove-widths there is no difference between the full-duplex system and the system with the triplex in the homo-purine region in both *continuous* and *isolated* cases. In the duplex regions the mean values for both minor and major grooves in the *isolated* cases are larger than in the *continuous* cases, whereas in the homo-purine region, not surprisingly, the major groove widens in comparison to the case where there is no Hoogsteen strand present. The major groove widening is, as argued previously, a result of local negative oversliding. The minor groove-widths are not notably affected by the presence of the anti-parallel third strand.

### Base pair flipping

**Figure 7.**
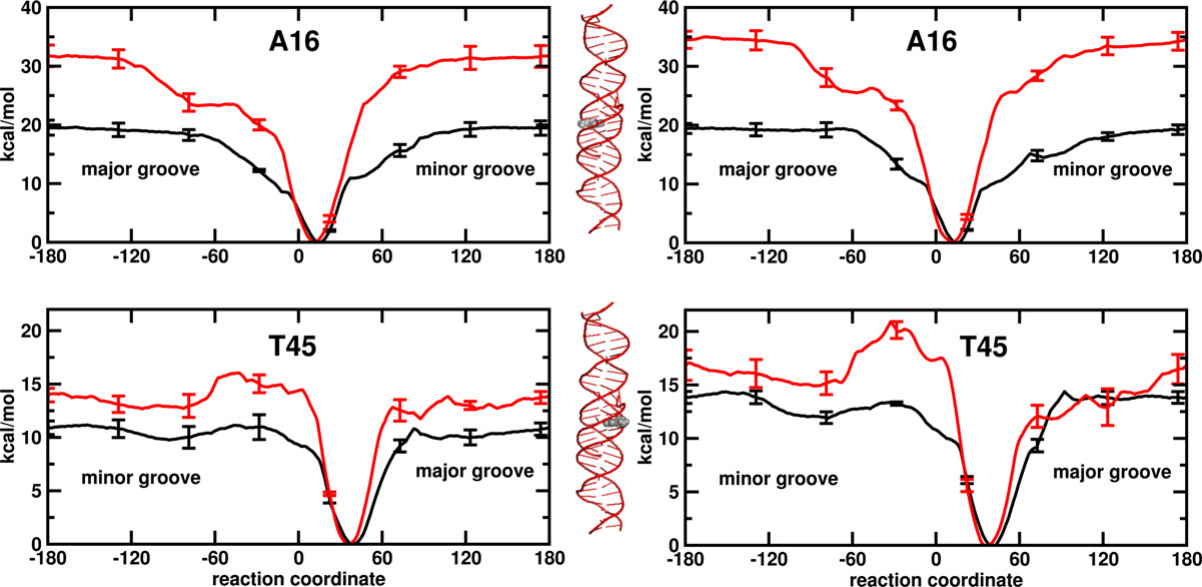
PMF for the flipping of the residues A16 and T45 as a function of the dihedral angle (°) used as reaction coordinate. Results for DNA helices as *isolated* molecules on the left side and as *continuous* molecules on the right side. Duplex and triplex in black and red. The labels major groove/minor groove indicate the flipping direction. Error bars were obtained by dividing data collection in each window to four parts and computing their standard deviation. The flipping residues are colored in grey in the 3D triple helical picture.

**Figure 8.**
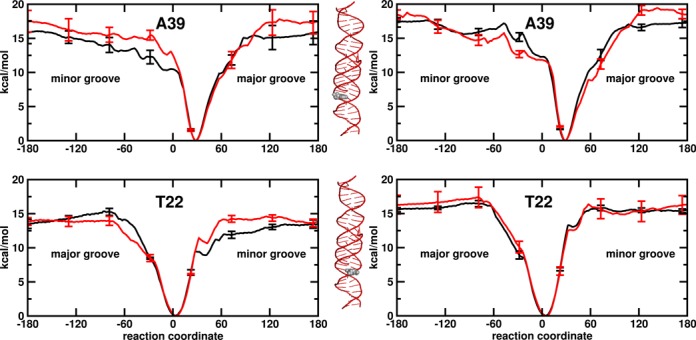
PMF for the flipping of the residues A39 and T22 as a function of the dihedral angle (°) used as reaction coordinate. Results for DNA helices as *isolated* molecules on the left side and as *continuous* molecules on the right side. Duplex and triplex in black and red. The labels major groove/minor groove indicate the flipping direction. Error bars were obtained by dividing data collection in each window to four parts and computing their standard deviation. The flipping residues are colored in grey in the 3D triple helical picture.

Free energy profiles for base flipping were calculated for base pairs A16·T45 and T22·A39. The two base pairs have a different position with respect to the TFO binding site. A16·T45 is in the binding site, while T22·A39 is one base pair away from the binding site. The free energy profiles for base flipping have been calculated independently for adenine and thymine flipping in the *isolated* and *continuous* DNA systems (Figures 7 and 8, left and right side, respectively). Interestingly, the profiles calculated in the *isolated* and *continuous* DNA systems do not show major difference. Below, first we report the results for the duplex case, then we discuss the effect of the TFO binding on the free energy profile.

In the case of full duplex system, the global minimum of the PMFs along the reaction coordinate corresponds to the Watson–Crick base pair state. The minimum is located around 28° and 12° for adenine and 3° and 38° for thymine for the T22·A39 and A16·T45 base pairs, respectively. The PMF curves rise quadratically as we move away from the minimum toward either the minor or the major groove. The flipped regions are relatively flat. Only in the case of A16·T45, a local minimum can be observed at around −90° in the thymine flipping surface toward minor groove. This results in a high barrier toward the minor groove opening with respect to the major groove. In the other cases the flipping barrier for the minor and major groove has the same height within the error margin. The results are consistent with experimental observations that both pathway are utilized by enzymes (63,64). In general, adenine has a slight high barrier than thymine in agreement with previous MD studies (65–67). For thymine opening, the barrier ranges from 11 to 17 kcal/mol and for adenine opening between 16 and 20 kcal/mol (see Materials and Methods section for definition of the energy barrier). Lemkul *et al.* (67) have reported a higher flipping barrier for adenine (at around 16 kcal/mol) than for cytosine (at around 13 kcal/mol) using the same reaction coordinate and a polarizable force field. Using a different reaction coordinate, Giudice *et al.* (65,66) have shown a higher barrier for adenine flipping than for thymine flipping.

The free energy changes for the flipping of thymine range between 6.7 and 8.5 kcal/mol, for the flipping of adenine slightly higher free energy values are observed ranging between 8.0 and 9.9 kcal/mol (see Materials and Methods section for details on how the free energy changes were calculated). NMR experiments report free energy changes of 5.5–7.2 kcal/mol (at 293 K) (68) and 5.7–7.3 kcal/mol (at 295.5 K) (69) for the opening of the adenine-thymine base pairs in diverse surrounding sequences. Our results also confirm the dependence of base pair opening on DNA sequence. A sequence effect in the range 0.8–1.0 kcal/mol in favor of the trimer CTC versus CTT is observed (Table 1). Interestingly, Coman and Russo (69) have observed a similar difference in free energy (0.6–0.9 kcal/mol) between the trimers TTT and TTC, but they have also reported a difference in free energy between two triple TTT, highlighting that sequence effects may be due to 4-mer effects or more (70). Sequence-dependence base pair opening can also be predicted using the statistical mechanism approach of Frank-Kamenetskii *et al.* (71,72). The approach is based on electrophoretic mobility data on DNA fragment and DNA melting data. Applying this set of parameters, an opposite trend is observed. The opening of the thymine-adenine base pair in trimer CTT is 0.4 kcal/mol more favorable than its opening in triple CTC. We have to note that this approach is based on the assumption that the event involves disruption of the base pair hydrogen bonds and base stacking interactions, leading to the flipping-out of both bases, while our approach releases one base at a time to an extra-helical position.

**Table 1.** Free energy changes (kcal/mol) in base pair opening for the base pairs A16·T45 and T22·A39 in the duplex and triplex DNA system (see Materials and Methods section for details on how the free energy changes were calculated)

	*isolated* DNA		*continuous* DNA	
A16·T45	A flipping	T flipping	A flipping	T flipping
Duplex	7.96	6.70	8.44	8.36
Triplex	15.50	9.59	19.79	8.43
T22·A39	A flipping	T flipping	A flipping	T flipping
Duplex	8.95	7.46	9.86	8.47
Triplex	8.88	7.86	8.33	8.99

For the *continuous* system the changes in free energy for base flipping in the duplex are on average 1 kcal/mol higher than for the *isolated* systems. The same observation is valid for the flipping barriers, which are one and up to 4 kcal/mol higher. Hindrance of the flipping process may be due to removal of non-intrinsic twisting and end-effects or it may be due to the restriction of helical bending. Indeed, previous theoretical studies showed a coupling between helical bending and base pair opening (66,73–74). But the effect on the calculated helical angle (see black curve in Figure 5) by making the molecules continuous is too small to make any conclusions as to the source of the difference.

When the TFO is bound to DNA, a shift of 2°–3° in the position of the minimum of PMFs is observed (Figures 7 and 8), indicating a tiny effect in the equilibrium geometry of the base pairs. The energy profile of the base pair located in the binding domain (homo-purine region) is clearly affected by the presence of the TFO (red profiles in Figure 7), while the opening of the base pair neighboring the binding domain is not (red profiles in Figure 8). Thus, both the barriers and the changes in free energy for A16·T45 opening are influenced by the presence of TFO. Upon binding, the barrier of thymine opening increases by around 5 kcal/mol, while the barrier of the adenine flipping increased by more than 10 kcal/mol. That indicates first a slower exchange between open and closed state in presence of TFO and, second, a slower exchange for adenine than for thymine. When adenine is hydrogen bonded to TFO via a reverse Hoogsteen base pair, a barrier of 16–20 kcal/mol in the thymine energy profile is observed between 30° and 60° toward the minor groove followed by a relative minimum at −90°, while for the adenine flipping a plateau around 25 kcal/mol is observed between 30° and 60°.

The free energy changes for thymine flipping range between 8.4 and 9.6 kcal/mol, which are slightly higher than for the duplex case, while for adenine a clear increase to 15.5–19.8 kcal/mol in free energy change is observed. The observed change in the barrier is reflected in a slow down of the opening process. Thus, the flipping of adenine is hindered in comparison to thymine flipping, both from the kinetic and thermodynamic point of view.

Based on the discussed results, we speculate how clamp oligonucleotides may work. We assume a clamp compound formed by two arms, a TFO arm and a Watson–Crick arm. First, the TFO arm binds to the DNA duplex in line with the observed mechanism for peptide nucleic acids (12). Then the Watson–Crick arm strand invades. Strand invasion will most likely take place once thymine flips in the minor groove in the duplex region, or in alternative when it flips in minor/major groove in triplex region.

## CONCLUSIONS

We have performed MD simulations to investigate a triplex forming DNA system at the atomistic level. We have focused on a highly stable anti-parallel triplex, formed by a purine-rich TFO targeting a purine-rich binding site of a DNA duplex and forming reverse Hoogsteen pairs with the Watson–Crick duplex. We have selected a double-strand DNA longer than the TFO and simulated it as duplex alone and in presence of the TFO in solution. Double-stranded DNA has been described as an isolated 30-mer helix or as a continuous helix to mimic two possible extreme cases. No major differences are observed in local structural properties and in relative base stability between the two descriptions. Only a small increase in the base flipping energy is observed by removing non-intrinsic twisting and end-effects.

The structural analysis shows that the purine-rich binding site on the DNA duplex is pre-conditionated to accommodate the Hoogsteen strand. Upon TFO binding, the double helix changes shape and becomes more rigid, the homo-purine region increases its major groove width mainly by oversliding in the negative direction. The obtained conformation is in a transition area between A and B conformations, which we refer to as B-like DNA conformations, whose base pairs remain almost perpendicular to the helical axis with non-zero Slide, reduced helical Twist and less deformable purine-purine steps. No significant change is observed in the neighboring duplex regions, these regions keep a B DNA conformation, and the base pairs breath with the same probability.

The local changes observed in the homo-purine region upon TFO binding, in particular regarding Slide, reflect a distortion of the whole helical structure, while changes in the variabilities of the base-step parameters, Slide and Roll, reflect a reduction of the total helical fluctuations. Interestingly, the change in global motion does not influence base pair stability in the duplex regions.

Opening of the A·T base pair located in the TFO binding site is less probable when the TFO is bound, while the A·T base pair located closer to the TFO binding site is not affected by the presence of the third strand. For the base pair located in the binding site, the presence of the TFO stabilizes the closed state (up to 7 kcal/mol) and increases the flipping barrier (up to 15 kcal/mol). In particular, the larger change in free energy is observed for the adenine involved in Watson–Crick pairing with the thymine of the DNA duplex, and the reverse Hoogsteen interaction with adenine of the TFO. The results suggest that strand invasion will more efficiently take place in the duplex region than in the triplex region, and will be most probably driven by thymine flipping into the minor groove.

The observed structural deformation and increase of rigidity of the helical conformation due to the presence of the TFO will cause inhibition of all those processes based on shape recognition (protein-DNA recognition via a lock-and-key approach) and/or those processes that require flexibility on the DNA helix (DNA binding via conformational selection mechanism). Since these conformational changes do not influence base breathing in the double strand region, processes, such as enzymes repair, acting close to the TFO site should not be affected by TFO binding. On the other hand, the TFO clearly hinders flipping of the base located on the purine strand of the binding site, both from a kinetic and thermodynamic point of view, making those bases less accessible to enzyme and strand invasion less efficient. The results support the notion that TFO binding can block the transcription machinery not only by blocking the initiation site, and/or altering DNA recognition by transcription factors, but also by blocking/slowing down the road to RNA polymerization by stabilizing base pairs in the target site.

## SUPPLEMENTARY DATA

Supplementary Data are available at NAR Online.

SUPPLEMENTARY DATA
