# Supplementary material for: Triple helical DNA in a duplex context and base pair opening
Source: Nucleic Acids Res. 2014 Sep 16;42(18):11329–38. doi: 10.1093/nar/gku848 (PMC4191418; doi:10.1093/nar/gku848)
Supplement: SUPPLEMENTARY DATA [file supp_42_18_11329__index.html]

Triple helical DNA in a duplex context and base pair opening — Triple helical DNA in a duplex context and base pair opening — SUPPLEMENTARY DATA 

# Triple helical DNA in a duplex context and base pair opening

## SUPPLEMENTARY DATA

**Files in this Data Supplement:**

- SUPPLEMENTARY DATA
