# Supplementary material for: Triple helical DNA in a duplex context and base pair opening
Source: Nucleic Acids Res. 2014 Sep 16;42(18):11329–38. doi: 10.1093/nar/gku848 (PMC4191418; doi:10.1093/nar/gku848)
Supplement: SUPPLEMENTARY DATA [file supp_gku848_nar-01945-f-2014-File005.pdf]

Triple helical DNA in a duplex context and base pair opening

Mauricio Esguerra*, Lennart Nilsson, Alessandra Villa†

August 15, 2014

Supplementary material

*Current address: Department of Cell and Molecular Biology, Uppsala University, Uppsala, Sweden
†Corresponding author

	inclination		x-disp.		Roll		Slide		zph		zp		overlap		major		minor		h-Twist		h-Rise	
step	mean	std	mean	std	mean	std	mean	std	mean	std	mean	std	mean	std	mean	std	mean	std	mean	std	mean	std
1	8.74	9.27	-0.66	1.23	5.42	5.80	0.09	0.52	0.82	1.39	-0.46	0.55	1.83	0.83					37.26	3.98	3.29	0.34
2	4.85	8.71	-1.04	1.19	2.80	5.31	-0.35	0.45	0.63	1.35	-0.09	0.50	3.45	1.30					36.50	4.51	3.26	0.34
3	11.54	8.98	-1.42	1.13	6.75	5.24	-0.21	0.41	1.81	1.40	0.09	0.52	2.43	0.95	15.97	1.50	13.74	0.99	35.01	3.90	3.13	0.35
4	15.11	10.54	-1.71	1.45	8.76	6.02	-0.17	0.46	2.36	1.53	0.15	0.46	0.37	0.46	17.04	1.55	13.92	0.93	35.18	4.19	3.13	0.38
5	3.71	7.48	-0.76	0.94	2.31	4.77	-0.28	0.35	0.90	1.12	0.36	0.45	3.29	1.11	16.94	1.51	13.91	0.94	38.59	3.89	3.27	0.29
6	9.40	9.79	-1.36	1.29	5.12	5.40	-0.25	0.43	1.62	1.60	0.21	0.59	2.79	1.00	17.30	1.52	13.83	0.97	32.74	4.15	3.01	0.34
7	11.47	12.05	-0.69	1.87	6.91	7.28	0.26	0.69	1.54	1.79	-0.11	0.49	0.39	0.57	16.86	1.40	13.36	1.01	37.84	4.25	3.27	0.39
8	12.43	8.99	-1.06	1.02	7.48	5.42	0.06	0.43	1.31	1.32	-0.51	0.56	2.21	0.83	16.65	1.50	13.11	1.14	36.03	3.70	3.27	0.31
9	16.22	11.71	-2.27	1.65	7.43	5.33	-0.17	0.45	2.01	1.86	-0.46	0.56	4.09	1.15	16.23	1.41	13.34	1.15	27.61	3.52	3.01	0.41
10	14.15	8.28	-1.84	1.06	9.91	5.83	-0.49	0.46	1.90	1.31	-0.10	0.69	1.20	0.85	16.94	1.38	13.47	1.10	41.78	4.20	3.31	0.34
11	3.28	7.36	-0.00	0.94	2.10	4.83	**0.20**	0.45	0.35	1.11	-0.14	0.45	2.01	0.83	17.48	1.29	13.63	0.97	39.41	3.67	3.26	0.27
12	9.96	9.62	-1.29	1.32	5.46	5.32	**-0.18**	0.42	1.52	1.58	0.02	0.54	2.82	1.02	16.82	1.64	13.87	0.88	32.79	4.04	3.03	0.34
13	7.26	8.26	-1.34	1.43	4.37	4.96	**-0.37**	0.54	1.40	1.26	0.33	0.45	2.22	1.12	16.57	1.50	13.69	0.82	36.31	4.47	3.34	0.34
14	10.06	8.14	-1.44	1.44	6.08	4.85	**-0.28**	0.56	1.64	1.28	0.16	0.49	2.32	1.03	16.90	1.55	13.36	0.84	36.11	4.52	3.32	0.33
15	4.31	7.33	-0.19	0.91	2.75	4.74	**0.13**	0.42	0.54	1.12	-0.10	0.43	2.16	0.84	16.52	1.52	13.54	0.86	39.00	3.79	3.23	0.27
16	11.62	9.57	-1.55	1.33	6.37	5.28	**-0.24**	0.44	1.85	1.56	0.09	0.56	2.74	1.02	16.40	1.61	13.94	0.89	32.95	4.03	3.03	0.37
17	9.06	8.76	-1.60	1.58	5.44	5.21	**-0.42**	0.60	1.70	1.39	0.38	0.53	2.17	1.15	16.31	1.57	13.93	0.91	36.34	4.71	3.32	0.37
18	10.96	8.44	-1.46	1.40	6.61	5.03	**-0.25**	0.56	1.72	1.31	0.11	0.54	2.45	1.09	16.85	1.68	13.54	0.94	36.03	4.45	3.30	0.33
19	4.65	7.20	-0.18	0.93	3.02	4.69	**0.16**	0.41	0.57	1.11	-0.11	0.44	2.03	0.80	16.45	1.52	13.36	0.87	39.37	3.66	3.25	0.26
20	5.85	8.63	-1.03	1.20	3.22	4.90	-0.26	0.41	0.76	1.42	-0.12	0.51	3.40	1.25	15.92	1.40	13.50	0.89	34.16	4.07	3.14	0.33
21	10.51	8.64	-1.35	1.11	6.44	5.30	-0.24	0.42	1.59	1.29	0.04	0.50	2.06	0.91	16.01	1.44	13.63	0.93	36.76	3.84	3.28	0.35
22	7.23	8.97	-0.79	1.10	4.29	5.41	-0.07	0.44	0.85	1.41	-0.24	0.51	2.11	0.83	16.60	1.44	13.67	0.96	36.11	3.95	3.19	0.31
23	22.00	11.47	-2.14	1.64	12.80	6.59	-0.03	0.54	3.14	1.66	-0.04	0.54	0.49	0.55	16.87	1.55	13.52	0.86	35.42	4.37	3.16	0.47
24	5.92	8.08	-0.38	1.02	3.77	5.21	0.10	0.45	0.81	1.23	-0.06	0.48	1.94	0.82	16.82	1.42	13.45	0.94	39.04	3.77	3.31	0.30
25	7.79	9.32	-1.62	1.21	4.15	5.01	-0.47	0.39	0.94	1.62	-0.24	0.62	3.93	1.16	17.28	1.56	13.72	1.09	32.12	3.63	3.06	0.35
26	15.96	10.11	-1.78	1.37	10.26	6.38	-0.22	0.53	2.57	1.42	0.30	0.50	0.26	0.38	16.87	1.46	14.12	1.09	38.87	4.19	3.35	0.45
27	8.02	9.93	-1.22	1.34	4.05	5.14	-0.23	0.42	1.23	1.62	0.02	0.62	3.71	1.23	17.65	1.59	14.70	1.38	31.78	4.57	2.98	0.35
28	7.68	12.54	-0.14	1.82	4.41	7.42	0.39	0.71	1.08	1.85	-0.03	0.55	0.58	0.83					37.29	5.04	3.19	0.40
29	-0.34	22.53	0.73	5.72	-0.69	13.52	0.12	1.28	0.08	2.83	0.11	1.17	2.10	2.19					30.37	28.31	3.49	1.28

Table S1: Isolated Duplex

	inclination		x-disp.		Roll		Slide		zph		zp		overlap		major		minor		h-Twist		h-Rise	
step	mean	std	mean	std	mean	std	mean	std	mean	std	mean	std	mean	std	mean	std	mean	std	mean	std	mean	std
1	7.81	8.11	-0.72	0.97	4.86	5.07	-0.01	0.39	0.86	1.24	-0.29	0.49	1.98	0.78					37.27	3.65	3.26	0.29
2	3.68	7.97	-0.71	1.08	2.12	4.88	-0.21	0.42	0.42	1.24	-0.13	0.47	3.02	1.24					36.41	4.17	3.24	0.30
3	9.24	8.60	-1.01	1.08	5.54	5.16	-0.10	0.39	1.38	1.38	0.00	0.53	2.44	0.92	15.71	1.30	13.42	0.87	35.90	3.75	3.19	0.33
4	13.92	9.72	-1.43	1.36	8.35	5.77	-0.09	0.47	2.18	1.44	0.15	0.45	0.27	0.37	16.56	1.34	13.51	0.82	36.16	4.03	3.19	0.36
5	3.65	7.07	-0.58	0.86	2.30	4.57	-0.18	0.34	0.82	1.10	0.29	0.43	3.00	1.08	16.35	1.36	13.56	0.83	39.02	3.77	3.26	0.29
6	8.59	9.06	-1.09	1.17	4.87	5.15	-0.16	0.43	1.35	1.44	0.06	0.55	2.57	0.98	16.68	1.28	13.66	0.87	33.98	4.09	3.09	0.32
7	12.01	11.51	-0.50	1.77	7.36	7.00	0.40	0.67	1.57	1.75	-0.16	0.49	0.36	0.53	16.39	1.14	13.35	0.90	38.05	4.11	3.26	0.39
8	10.87	7.88	-0.81	0.94	6.72	4.86	0.11	0.40	1.09	1.16	-0.51	0.47	1.97	0.76	16.12	1.29	12.99	0.89	36.91	3.69	3.31	0.28
9	8.53	7.88	-0.68	0.90	5.38	5.00	0.05	0.39	0.88	1.15	-0.38	0.45	1.88	0.78	15.26	1.17	12.96	0.80	37.61	3.70	3.23	0.29
10	12.27	8.73	-1.17	1.12	7.16	5.08	-0.02	0.39	1.68	1.43	-0.15	0.54	2.55	1.03	15.52	1.19	12.96	0.76	34.81	3.84	3.17	0.33
11	2.16	6.89	0.19	0.81	1.43	4.77	**0.26**	0.38	0.22	1.03	-0.10	0.40	1.61	0.76	15.82	1.13	13.09	0.77	40.81	3.48	3.29	0.25
12	7.89	8.51	-0.89	1.14	4.49	4.85	**-0.08**	0.39	1.09	1.36	-0.11	0.47	2.58	0.99	16.06	1.29	13.37	0.79	33.80	3.55	3.11	0.31
13	6.54	7.47	-0.78	1.10	4.14	4.71	**-0.11**	0.47	1.15	1.11	0.20	0.43	2.29	1.12	15.64	1.22	13.31	0.73	37.78	3.93	3.36	0.29
14	9.92	7.67	-0.98	1.16	6.17	4.73	**-0.04**	0.46	1.48	1.19	0.03	0.47	2.26	1.05	16.20	1.30	13.06	0.75	36.97	3.99	3.34	0.28
15	3.90	6.98	0.06	0.84	2.55	4.63	**0.27**	0.37	0.36	1.07	-0.21	0.41	1.95	0.82	15.76	1.24	13.20	0.81	39.61	3.61	3.23	0.26
16	10.35	8.94	-1.19	1.20	5.80	5.02	**-0.12**	0.40	1.52	1.47	-0.05	0.51	2.60	1.00	15.64	1.33	13.54	0.79	33.58	3.78	3.07	0.33
17	7.29	7.50	-0.94	1.09	4.66	4.79	**-0.18**	0.45	1.30	1.12	0.24	0.43	2.28	1.13	15.69	1.24	13.52	0.79	38.04	4.09	3.37	0.31
18	10.62	7.97	-1.09	1.23	6.50	4.86	**-0.06**	0.50	1.59	1.25	0.02	0.49	2.36	1.10	16.21	1.34	13.24	0.82	36.49	4.11	3.30	0.29
19	4.27	6.74	-0.02	0.81	2.83	4.53	**0.24**	0.37	0.47	1.03	-0.15	0.41	1.95	0.78	15.88	1.30	13.17	0.81	40.07	3.46	3.27	0.24
20	4.66	8.08	-0.73	1.12	2.59	4.64	-0.16	0.41	0.53	1.34	-0.17	0.50	2.97	1.21	15.57	1.24	13.29	0.89	34.72	3.99	3.14	0.30
21	10.18	8.08	-1.17	1.01	6.34	5.04	-0.16	0.40	1.51	1.25	0.00	0.49	2.12	0.90	15.66	1.28	13.39	0.91	37.14	3.71	3.29	0.33
22	6.59	8.41	-0.60	1.02	3.99	5.17	0.01	0.42	0.70	1.32	-0.28	0.49	2.03	0.79	16.13	1.32	13.47	0.89	36.71	3.85	3.21	0.29
23	20.81	10.70	-1.82	1.59	12.33	6.31	0.09	0.55	2.93	1.59	-0.09	0.54	0.44	0.52	16.55	1.38	13.36	0.79	35.98	4.35	3.22	0.43
24	5.83	7.70	-0.25	0.99	3.79	5.03	0.18	0.44	0.77	1.19	-0.07	0.47	1.85	0.82	16.44	1.28	13.30	0.86	39.56	3.68	3.31	0.28
25	6.46	8.65	-1.33	1.12	3.48	4.72	-0.39	0.37	0.67	1.53	-0.31	0.59	3.71	1.17	16.61	1.43	13.51	0.98	32.49	3.53	3.07	0.32
26	17.00	9.67	-1.66	1.27	11.12	6.20	-0.11	0.50	2.62	1.36	0.21	0.49	0.22	0.34	15.97	1.29	13.61	0.95	39.47	3.94	3.36	0.44
27	6.01	8.93	-0.94	1.20	3.19	4.87	-0.20	0.41	0.81	1.46	-0.09	0.58	3.44	1.22	17.14	1.37	13.72	1.02	33.27	4.22	3.08	0.33
28	8.19	10.16	-0.44	1.42	5.28	6.61	0.19	0.60	1.29	1.49	0.13	0.49	0.23	0.39					39.70	3.65	3.29	0.35
29	8.91	10.02	-1.63	1.26	4.45	5.07	-0.39	0.40	1.21	1.67	-0.13	0.69	4.17	1.18					30.82	4.00	2.99	0.35

Table S2: Continuous Duplex

	inclination		x-disp.		Roll		Slide		zph		zp		overlap		major		minor		h-Twist		h-Rise	
step	mean	std	mean	std	mean	std	mean	std	mean	std	mean	std	mean	std	mean	std	mean	std	mean	std	mean	std
1	9.36	9.52	-0.71	1.19	5.82	6.04	0.09	0.52	0.95	1.40	-0.41	0.54	1.84	0.84					37.39	4.20	3.27	0.35
2	4.50	8.79	-1.01	1.17	2.58	5.28	-0.35	0.44	0.58	1.36	-0.09	0.52	3.52	1.28					36.32	4.32	3.26	0.34
3	10.79	9.00	-1.38	1.18	6.32	5.29	-0.22	0.42	1.72	1.42	0.11	0.54	2.34	0.93	16.02	1.52	13.80	1.03	35.12	3.98	3.16	0.35
4	15.14	10.60	-1.68	1.43	8.76	5.98	-0.16	0.45	2.43	1.58	0.22	0.49	0.37	0.44	17.06	1.55	13.85	0.90	35.11	4.04	3.10	0.40
5	2.32	7.08	-0.61	0.91	1.41	4.62	-0.26	0.35	0.70	1.09	0.37	0.44	3.16	1.11	16.84	1.51	13.81	0.88	38.87	3.87	3.32	0.29
6	9.43	10.02	-1.23	1.30	5.19	5.52	-0.18	0.44	1.58	1.64	0.17	0.57	2.76	0.98	17.07	1.47	13.73	0.89	33.28	4.08	3.02	0.35
7	10.36	12.04	-0.52	1.93	6.20	7.27	0.32	0.72	1.35	1.80	-0.14	0.49	0.42	0.59	16.92	1.40	13.13	0.99	37.65	4.33	3.27	0.39
8	11.79	8.83	-0.96	1.06	7.16	5.31	0.08	0.44	1.24	1.30	-0.48	0.56	2.08	0.82	16.59	1.53	12.57	1.28	36.61	3.80	3.29	0.31
9	12.31	11.15	-1.89	1.58	5.81	5.25	-0.22	0.45	1.34	1.76	-0.55	0.52	3.78	1.09	15.96	1.44	12.72	1.26	28.10	3.43	3.10	0.37
10	14.97	7.89	-1.82	0.95	10.66	5.65	-0.46	0.44	1.90	1.26	-0.20	0.67	1.17	0.72	16.74	1.43	13.01	1.08	42.52	4.17	3.34	0.39
11	2.15	8.08	0.20	1.08	1.33	5.23	**0.28**	0.56	0.13	1.24	-0.19	0.45	2.10	1.07	17.61	1.33	13.50	0.92	39.11	4.20	3.20	0.28
12	11.44	8.57	-2.09	1.30	6.01	4.55	**-0.49**	0.44	1.71	1.38	-0.04	0.51	2.97	0.95	18.23	1.40	14.17	0.81	31.17	4.05	2.97	0.34
13	11.45	8.09	-2.86	1.25	6.37	4.50	**-0.96**	0.43	2.21	1.18	0.51	0.44	2.80	0.89	18.34	1.25	14.03	0.76	32.93	3.83	3.08	0.35
14	14.39	7.40	-3.69	1.38	7.80	4.09	**-1.15**	0.42	2.47	1.09	0.33	0.40	2.51	0.93	19.74	1.35	13.51	0.72	31.95	4.23	3.17	0.35
15	4.97	6.22	-0.53	0.71	3.29	4.12	**-0.09**	0.34	0.79	0.96	0.06	0.39	2.61	0.78	19.41	0.92	13.46	0.62	39.30	3.03	3.09	0.26
16	11.49	9.00	-3.33	1.20	5.57	4.39	**-0.99**	0.34	1.85	1.39	0.10	0.46	2.70	0.78	19.17	0.94	13.79	0.67	28.31	3.29	3.04	0.38
17	13.16	7.18	-2.61	1.10	7.73	4.22	**-0.82**	0.39	2.33	1.07	0.39	0.40	2.42	0.85	18.80	0.92	13.94	0.69	34.81	3.69	3.10	0.31
18	15.81	7.85	-3.54	1.36	8.57	4.26	**-1.03**	0.40	2.56	1.13	0.21	0.40	2.47	0.85	19.26	0.98	13.72	0.75	32.18	4.05	3.08	0.36
19	5.64	6.73	-0.53	0.82	3.62	4.29	**-0.04**	0.37	0.89	1.10	0.06	0.43	2.34	0.80	18.36	1.01	13.62	0.75	38.62	3.30	3.15	0.24
20	6.40	8.86	-1.41	1.20	3.45	4.88	-0.45	0.42	0.98	1.39	0.01	0.51	3.65	1.26	17.30	1.33	13.76	1.00	33.67	4.11	3.10	0.32
21	11.77	9.02	-1.47	1.15	7.16	5.52	-0.24	0.43	1.82	1.41	0.09	0.55	2.64	1.00	16.02	1.43	13.89	0.99	36.21	3.81	3.21	0.35
22	8.65	9.17	-0.83	1.06	5.22	5.55	-0.02	0.41	0.97	1.43	-0.31	0.50	2.09	0.86	16.28	1.45	13.90	0.95	36.61	4.14	3.23	0.32
23	22.54	11.08	-2.22	1.61	13.12	6.40	-0.03	0.53	3.15	1.58	-0.12	0.52	0.47	0.54	16.78	1.39	13.60	0.87	35.47	4.48	3.17	0.45
24	5.99	8.01	-0.31	1.05	3.85	5.18	0.15	0.45	0.82	1.24	-0.05	0.47	1.93	0.82	16.80	1.35	13.42	0.94	39.34	3.71	3.31	0.29
25	6.45	9.24	-1.38	1.22	3.40	4.98	-0.41	0.39	0.67	1.56	-0.31	0.56	3.83	1.19	17.26	1.52	13.62	1.06	32.18	3.65	3.05	0.33
26	17.02	9.99	-1.84	1.36	10.89	6.31	-0.20	0.51	2.63	1.38	0.20	0.48	0.25	0.36	16.56	1.39	13.71	1.01	38.58	3.98	3.31	0.43
27	6.23	9.38	-1.07	1.29	3.23	5.05	-0.25	0.43	0.88	1.48	-0.06	0.58	3.70	1.27	17.41	1.62	13.81	1.11	32.94	4.48	3.07	0.34
28	9.16	11.65	-0.56	1.69	5.69	7.29	0.19	0.68	1.40	1.73	0.10	0.52	0.31	0.46					38.77	4.06	3.24	0.39
29	7.46	12.21	-1.28	1.76	3.58	6.47	-0.25	0.62	0.86	1.94	-0.25	0.75	3.74	1.44					31.87	4.98	3.02	0.41

Table S3: Isolated Triplex

	inclination		x-disp.		Roll		Slide		zph		zp		overlap		major		minor		h-Twist		h-Rise	
step	mean	std	mean	std	mean	std	mean	std	mean	std	mean	std	mean	std	mean	std	mean	std	mean	std	mean	std
1	7.14	7.55	-0.48	0.95	4.53	4.79	0.11	0.40	0.68	1.13	-0.36	0.46	1.84	0.75					38.01	3.49	3.30	0.27
2	2.11	7.60	-0.25	1.09	1.17	4.78	-0.02	0.46	0.11	1.24	-0.21	0.48	2.47	1.23					37.25	3.97	3.24	0.30
3	6.61	8.28	-0.58	1.04	4.04	5.14	0.02	0.40	0.89	1.31	-0.10	0.50	2.26	0.88	15.35	1.14	12.98	0.83	36.66	3.74	3.24	0.32
4	10.86	9.01	-0.83	1.26	6.72	5.52	0.11	0.45	1.67	1.34	0.08	0.41	0.24	0.37	16.24	1.23	13.14	0.79	37.27	3.88	3.26	0.31
5	2.38	6.45	-0.36	0.84	1.51	4.27	-0.10	0.35	0.61	1.02	0.26	0.41	2.65	1.07	16.12	1.22	13.20	0.80	39.26	3.75	3.29	0.26
6	6.70	8.50	-0.65	1.12	3.85	4.94	-0.01	0.40	0.98	1.38	-0.03	0.50	2.56	0.92	16.31	1.22	13.19	0.81	34.68	3.86	3.09	0.30
7	8.88	9.89	0.03	1.54	5.69	6.31	0.57	0.63	0.99	1.49	-0.28	0.43	0.30	0.48	16.18	1.11	12.73	0.85	39.47	3.69	3.35	0.33
8	12.51	7.63	-0.71	0.88	7.81	4.71	0.26	0.37	1.22	1.15	-0.59	0.50	1.96	0.77	15.89	1.17	12.63	0.93	37.27	3.46	3.29	0.28
9	11.15	9.84	-1.21	1.31	5.50	4.89	0.02	0.38	1.05	1.54	-0.68	0.45	3.65	1.15	15.43	1.12	12.83	0.92	29.46	3.17	3.16	0.30
10	11.34	7.11	-1.41	0.92	7.96	5.02	-0.35	0.43	1.48	1.04	-0.15	0.42	1.28	0.77	16.17	1.19	12.45	0.90	41.72	3.44	3.33	0.31
11	3.99	6.14	0.26	0.73	2.79	4.31	**0.41**	0.37	0.33	0.94	-0.25	0.39	1.51	0.62	16.32	1.29	12.65	0.80	41.43	3.42	3.27	0.24
12	10.60	7.25	-0.69	0.95	6.32	4.33	**0.15**	0.38	1.20	1.13	-0.40	0.44	2.79	0.98	15.87	1.06	13.56	0.68	35.28	3.15	3.05	0.30
13	12.01	7.63	-2.17	1.39	6.90	4.34	**-0.56**	0.50	1.97	1.14	0.20	0.41	3.27	1.01	16.06	0.87	13.59	0.70	34.23	4.49	3.16	0.34
14	13.87	7.67	-2.82	1.25	7.80	4.33	**-0.81**	0.42	2.21	1.13	0.14	0.42	2.17	0.88	18.29	1.42	13.14	0.66	33.32	3.95	3.15	0.33
15	4.08	6.40	-0.40	0.70	2.70	4.28	**-0.05**	0.32	0.61	0.99	0.01	0.39	2.86	0.73	18.87	1.21	13.17	0.64	39.54	2.87	3.11	0.27
16	11.24	8.83	-3.07	1.16	5.59	4.40	**-0.93**	0.34	1.93	1.44	0.21	0.51	2.53	0.77	18.58	0.95	13.57	0.65	29.07	3.14	3.02	0.39
17	11.46	7.15	-2.39	1.01	6.75	4.23	**-0.78**	0.37	2.07	1.06	0.37	0.45	2.47	0.82	18.53	0.87	13.90	0.64	34.83	3.70	3.15	0.30
18	15.59	8.13	-3.20	1.26	8.72	4.54	**-0.93**	0.38	2.55	1.22	0.25	0.41	2.37	0.85	18.80	0.94	13.70	0.71	33.19	3.94	3.06	0.36
19	4.53	6.30	-0.41	0.78	2.96	4.14	**-0.02**	0.35	0.68	0.99	0.01	0.39	2.15	0.74	17.87	0.93	13.31	0.73	39.22	3.26	3.19	0.24
20	2.94	7.92	-0.73	1.14	1.62	4.73	-0.26	0.42	0.36	1.25	-0.08	0.45	3.08	1.22	16.72	1.02	13.08	0.86	35.27	3.84	3.16	0.28
21	8.32	8.54	-0.81	1.05	5.22	5.42	-0.04	0.40	1.15	1.29	-0.08	0.45	2.32	0.99	15.36	1.16	13.08	0.86	37.51	3.56	3.27	0.33
22	6.51	8.32	-0.23	1.01	3.94	5.17	0.23	0.41	0.45	1.25	-0.52	0.47	2.03	0.81	15.46	1.18	13.12	0.87	37.04	3.75	3.24	0.28
23	17.12	10.09	-1.09	1.51	10.74	6.23	0.32	0.55	2.27	1.50	-0.20	0.50	0.31	0.47	15.90	1.30	12.85	0.74	37.87	4.10	3.38	0.38
24	4.22	6.94	0.05	0.88	2.79	4.69	0.29	0.42	0.44	1.07	-0.17	0.46	1.76	0.74	16.08	1.18	12.74	0.77	40.25	3.47	3.30	0.27
25	4.37	8.48	-0.87	1.15	2.32	4.70	-0.25	0.38	0.35	1.50	-0.32	0.55	3.20	1.24	16.24	1.26	12.97	0.90	33.16	3.56	3.07	0.30
26	13.88	8.90	-1.16	1.18	9.44	6.00	0.04	0.48	2.09	1.23	0.12	0.44	0.15	0.30	15.59	1.13	13.04	0.84	40.65	3.69	3.49	0.36
27	2.92	7.85	-0.35	1.15	1.55	4.54	-0.03	0.42	0.21	1.31	-0.23	0.54	2.91	1.22	16.69	1.22	13.04	0.92	34.25	4.15	3.12	0.29
28	5.86	9.22	0.11	1.32	3.90	6.32	0.44	0.59	0.80	1.38	-0.04	0.46	0.20	0.39					41.12	3.35	3.31	0.31
29	6.94	9.37	-1.11	1.27	3.48	4.83	-0.21	0.43	0.68	1.50	-0.38	0.57	3.57	1.30					31.44	3.88	3.07	0.31

Table S4: Continuous Triplex

	inclination		x-disp.		Roll		Slide		zph		zp		overlap		major		minor		h-Twist		h-Rise	
step	mean	std	mean	std	mean	std	mean	std	mean	std	mean	std	mean	std	mean	std	mean	std	mean	std	mean	std
61	0.78	9.13	0.07	1.41	0.40	5.84	0.12	0.55	0.19	1.38	0.01	0.41	3.36	1.93					38.16	4.14	3.39	0.28
62	0.84	7.70	0.38	1.24	0.44	4.29	0.26	0.44	-0.03	1.23	0.04	0.36	2.89	1.01					32.97	3.74	3.12	0.25
63	-2.76	6.83	1.08	1.10	-1.59	3.88	0.48	0.40	-0.64	1.12	0.17	0.36	2.87	0.90	15.39	0.79	22.04	0.37	33.57	3.81	3.05	0.27
64	-6.08	8.66	2.08	1.37	-2.89	4.09	0.71	0.42	-0.77	1.27	0.58	0.36	2.76	0.75	15.41	0.68	21.82	0.45	28.40	3.53	2.87	0.37
65	-4.97	5.91	-0.32	0.75	-3.57	4.22	-0.48	0.36	-0.38	0.84	0.25	0.38	5.49	1.04	15.92	0.76	22.20	0.57	41.73	3.21	3.24	0.17
66	-5.52	7.83	1.56	1.51	-2.83	4.10	0.56	0.57	-1.09	1.28	-0.10	0.49	2.73	1.17	16.53	0.86	22.78	0.46	31.66	3.86	2.88	0.42
67	-2.40	7.33	0.24	1.66	-1.07	3.45	-0.04	0.61	-0.67	1.18	-0.45	0.62	5.17	1.77	17.15	1.06	22.38	0.51	28.97	4.83	3.13	0.23
68	-24.64	43.39	0.48	1.36	40.12	63.45	1.96	1.31	-2.15	3.53	-1.18	1.00	0.21	0.71					49.19	150.73	1.70	1.91
69	4.25	23.75	-0.48	3.26	1.54	31.22	-0.19	1.46	-3.33	1.83	-3.78	1.96	0.69	1.32					-0.22	123.38	-1.11	1.07

Table S5: Average parameters for Hoogsteen base pair steps in the *continuous* simulation.

	inclination		x-disp.		Roll		Slide		zph		zp		overlap		major		minor		h-Twist		h-Rise	
step	mean	std	mean	std	mean	std	mean	std	mean	std	mean	std	mean	std	mean	std	mean	std	mean	std	mean	std
61	-1.14	8.62	0.13	1.30	-0.76	5.47	0.03	0.58	-0.15	1.32	-0.04	0.42	3.93	2.08					37.44	4.12	3.34	0.27
62	0.82	7.68	0.29	1.24	0.40	4.26	0.22	0.44	-0.10	1.20	0.02	0.40	2.94	0.95					32.82	3.94	3.15	0.26
63	-3.24	6.75	1.16	1.18	-1.82	3.76	0.48	0.45	-0.73	1.10	0.14	0.39	2.81	0.94	15.31	0.75	22.12	0.46	32.94	3.45	3.02	0.29
64	-4.18	9.26	1.98	1.45	-1.96	4.24	0.72	0.39	-0.68	1.30	0.42	0.43	2.82	0.73	14.90	0.70	22.01	0.57	27.51	3.84	2.93	0.35
65	-5.74	5.87	0.05	0.88	-4.13	4.19	-0.26	0.45	-0.48	0.86	0.32	0.38	4.62	1.45	15.03	0.77	22.25	0.55	41.76	3.66	3.24	0.19
66	-2.09	7.51	0.21	1.48	-1.19	4.09	0.02	0.57	-0.32	1.25	0.06	0.39	3.26	1.17	15.38	0.81	22.28	0.76	31.98	4.05	3.12	0.29
67	0.16	6.80	0.44	1.37	0.12	3.64	0.23	0.56	-0.10	1.12	0.21	0.52	3.33	1.06	15.79	1.02	20.63	1.29	32.07	3.96	3.11	0.24
68	-22.94	31.22	1.18	1.46	-12.44	64.83	1.85	1.55	-2.32	3.16	-0.36	1.94	0.52	1.19					98.30	93.32	2.70	2.20
69	10.97	36.19	0.13	2.14	26.33	33.67	0.63	1.06	-0.69	5.39	-1.01	3.52	1.31	1.69					19.03	97.77	-1.00	1.43

Table S6: Average parameters for Hoogsteen base pair steps in the *isolated* simulation.

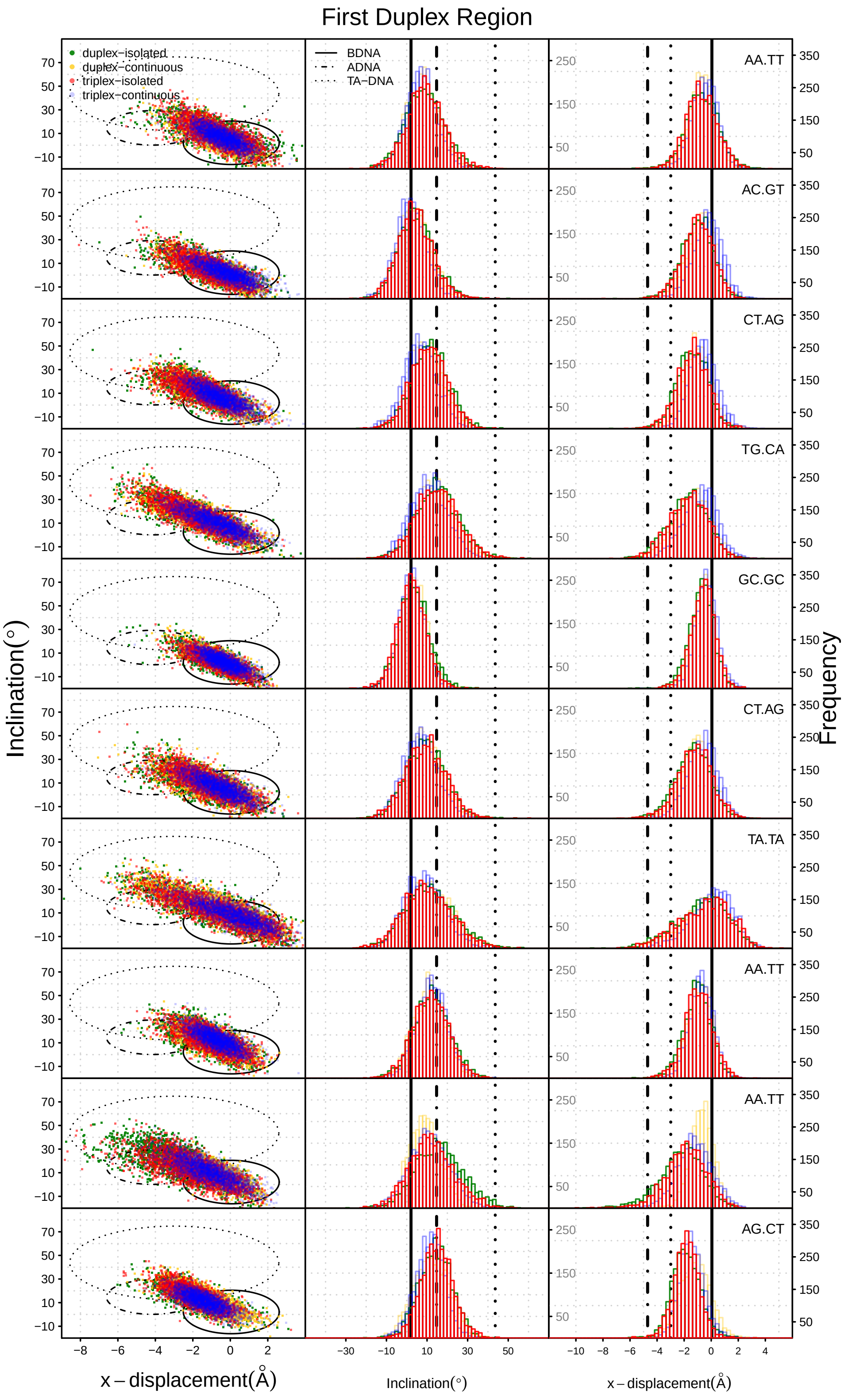

Figure S1: Local helical parameters Inclination vs. x-displacement for the first duplex region. Ellipse axes have a radius of twice the standard deviations given in Table 2 of Lu and Olson? .

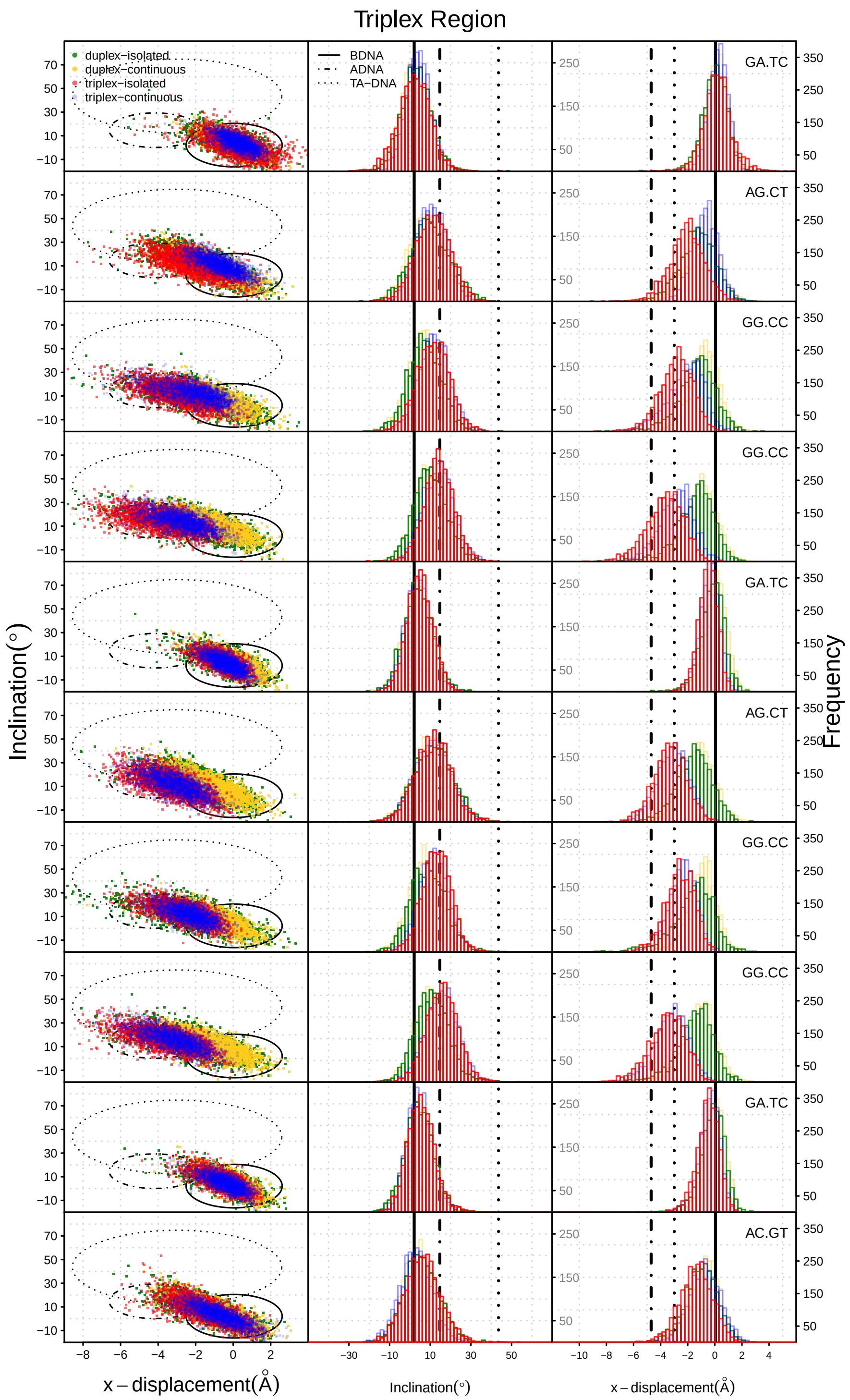

Figure S2: Local helical parameters Inclination vs. X-displacement for each base pair step in the triplex bound region of the simulated DNA. Notice the change from duplex to Hoogsteen bound triplex.

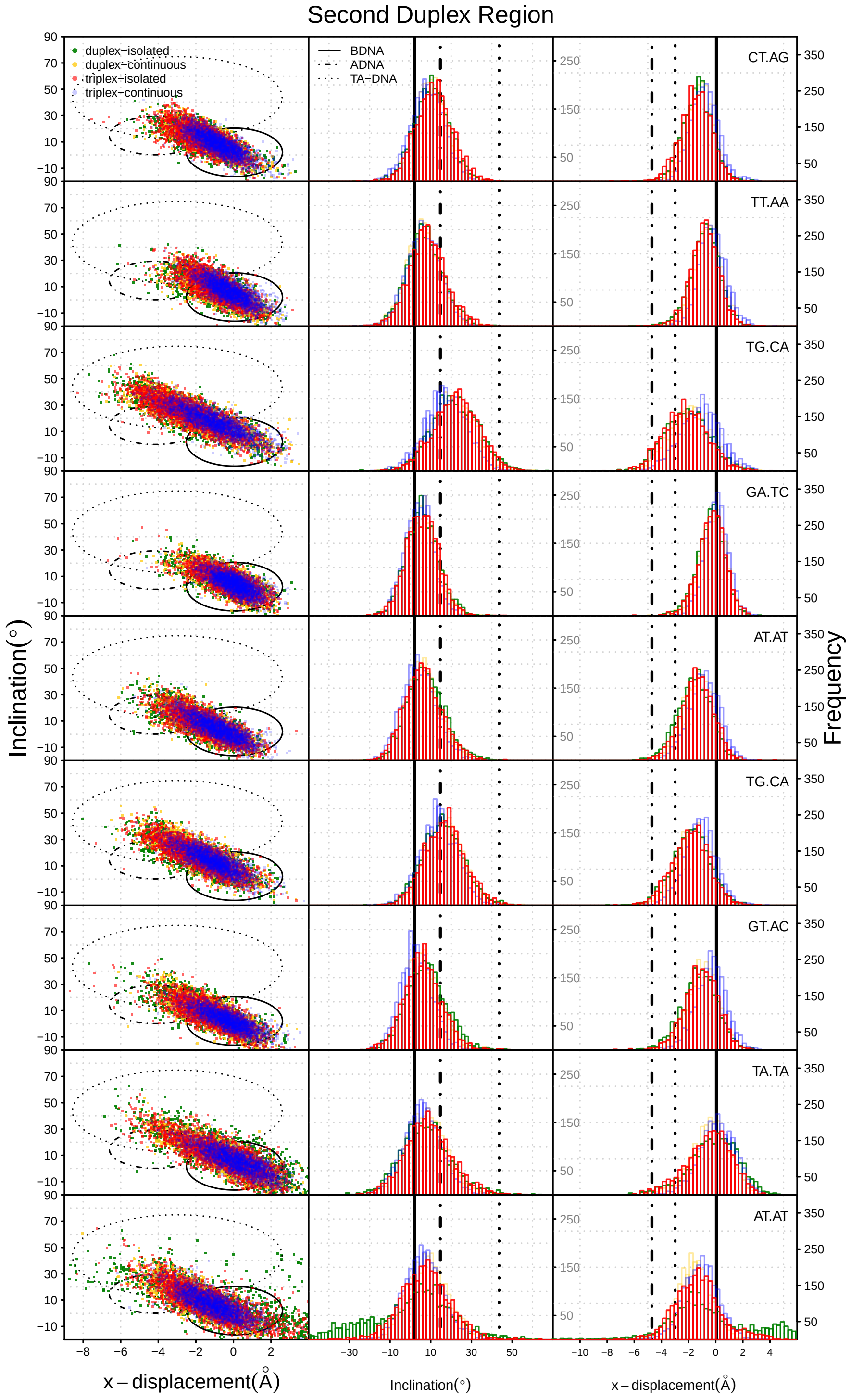

Figure S3: Local helical parameters Inclination vs. X-displacement for the second double-stranded helical region of the modeled DNA.

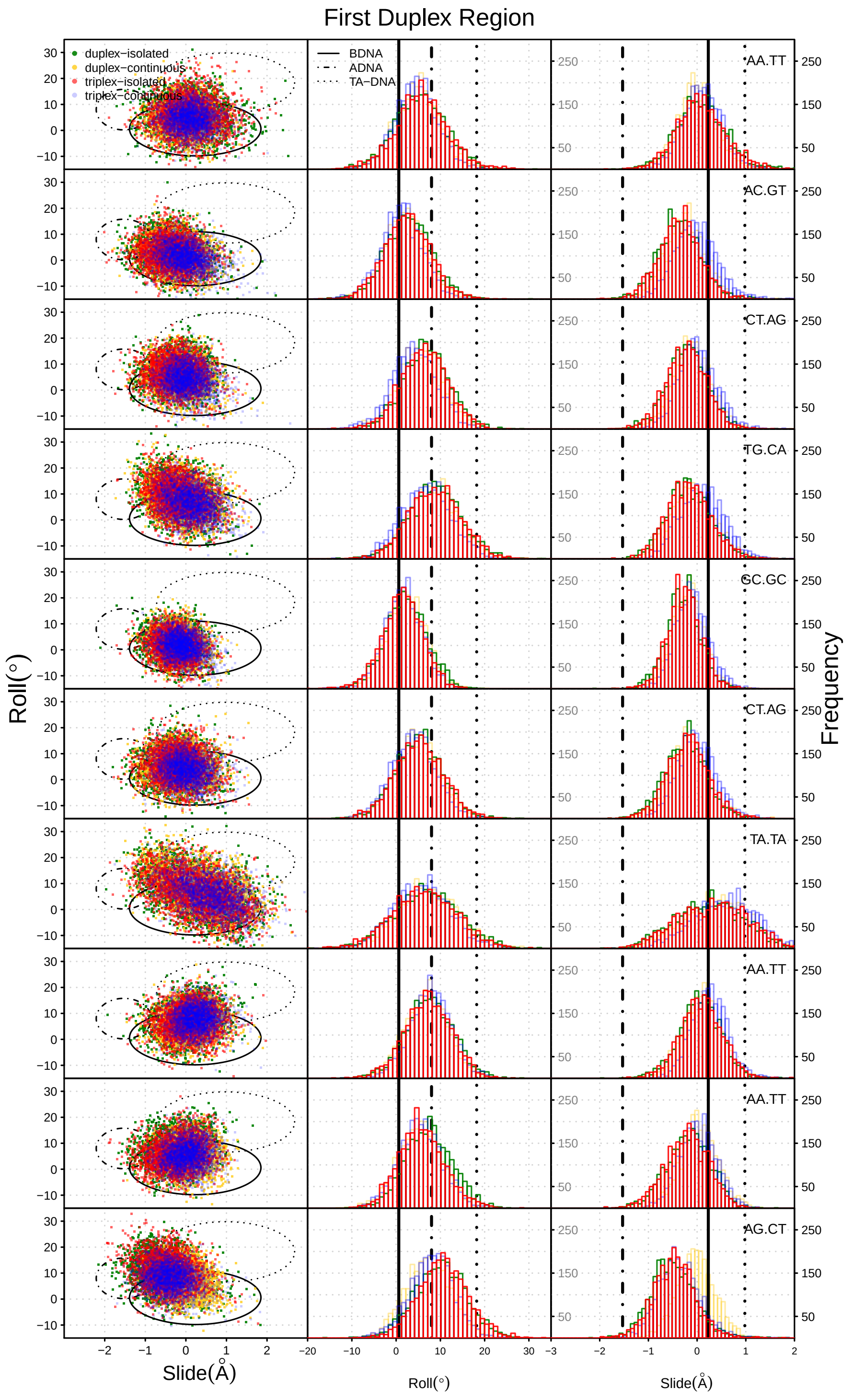

Figure S4: Base pair step parameters Roll vs. Slide for the first double-stranded helical region of the modeled DNA.

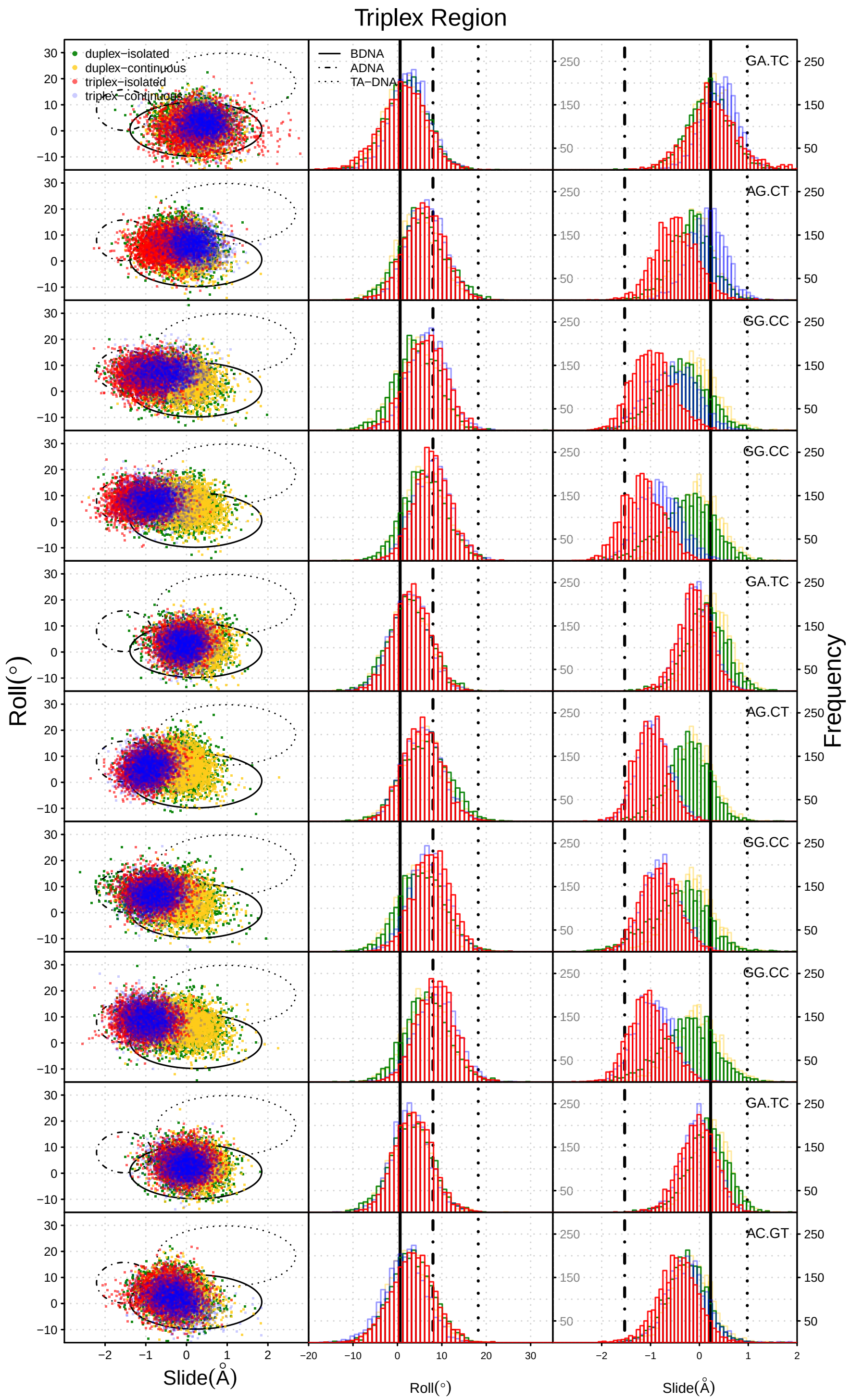

Figure S5: Base pair step parameters Roll vs. Slide for the triple-stranded helical region of the modeled DNA.

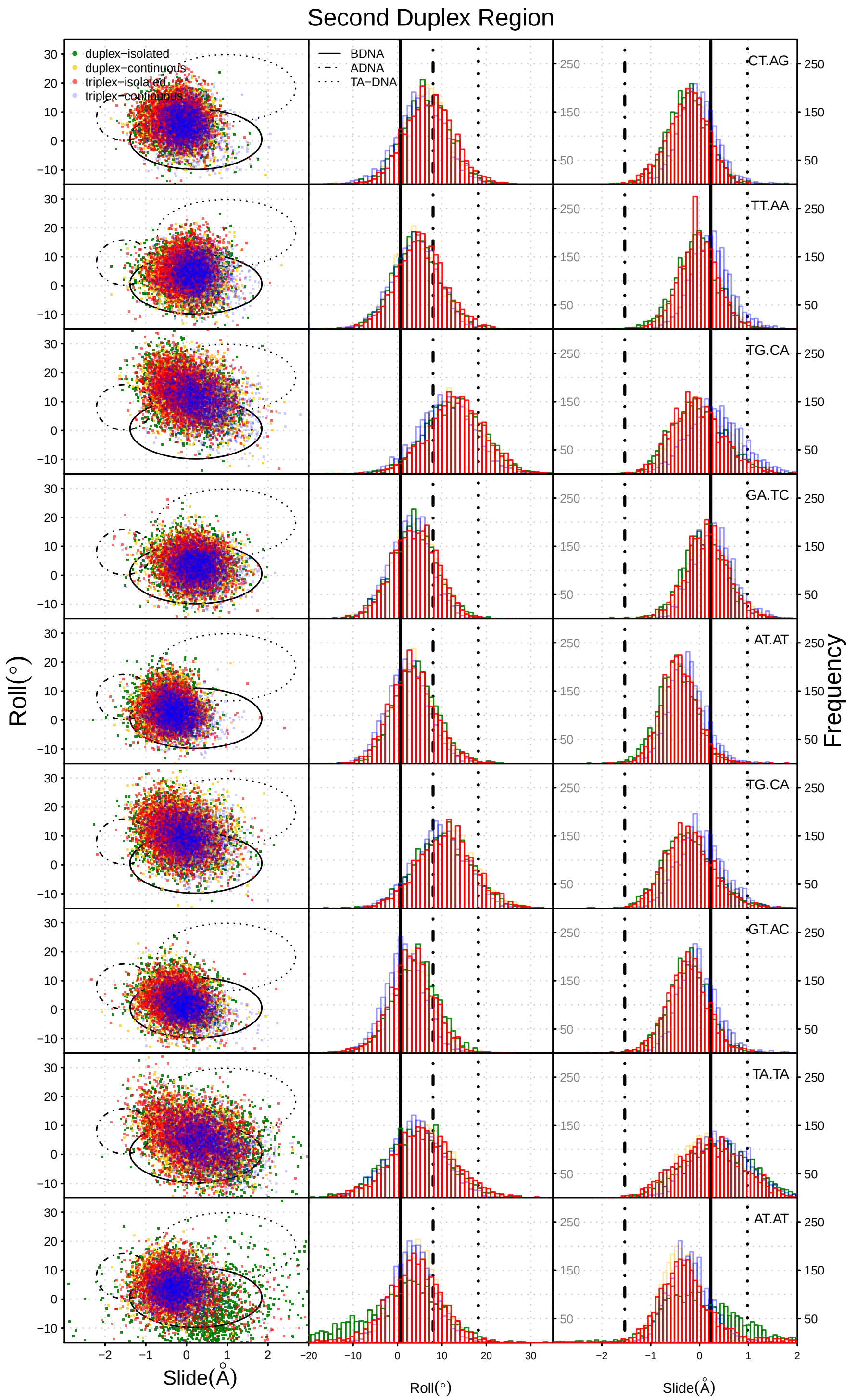

Figure S6: Base pair step parameters Roll vs. Slide for the triple-stranded helical region of the modeled DNA.

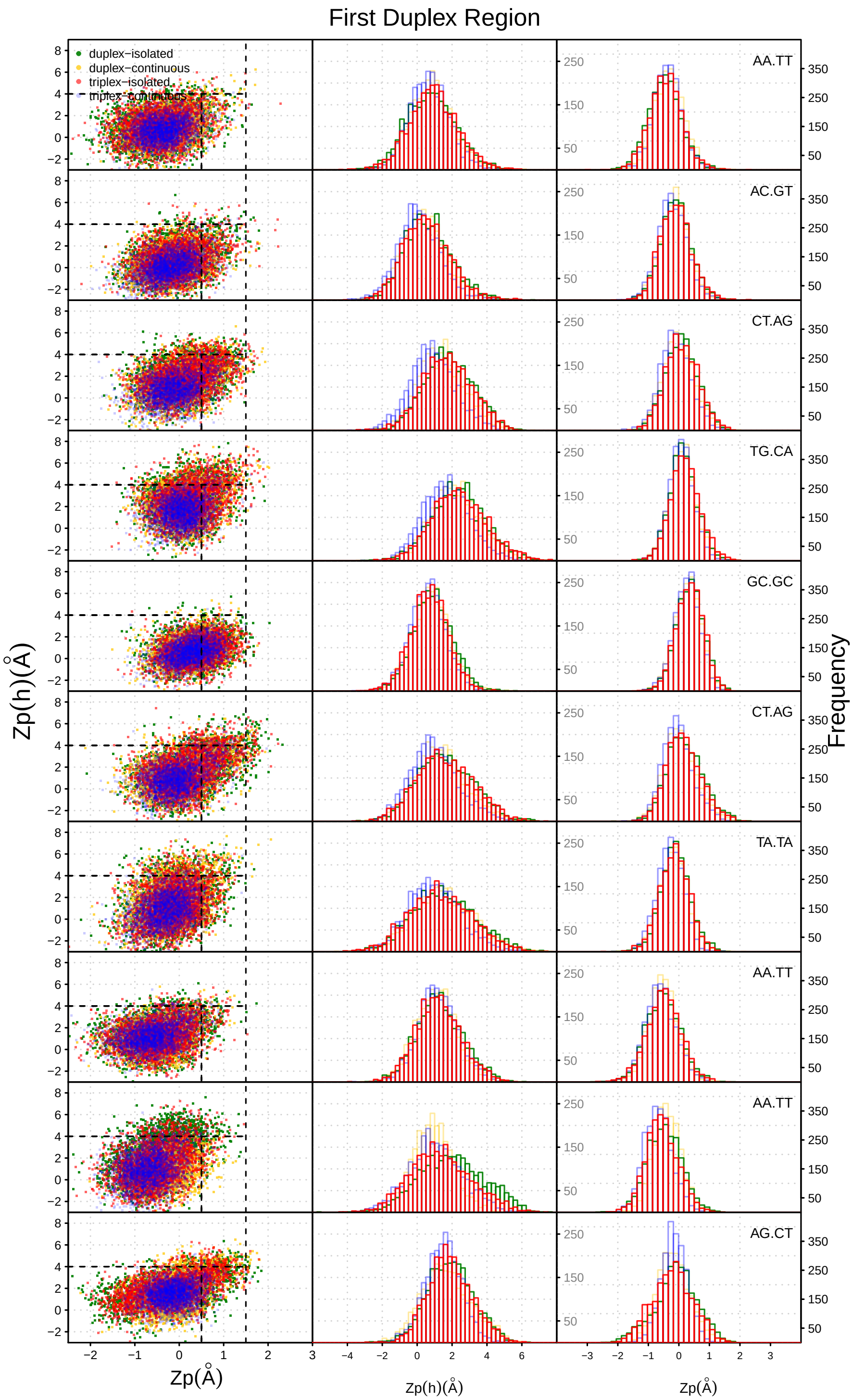

Figure S7: Inter-strand p to p vector projections on z axis of lhp reference frame and bps reference frame.

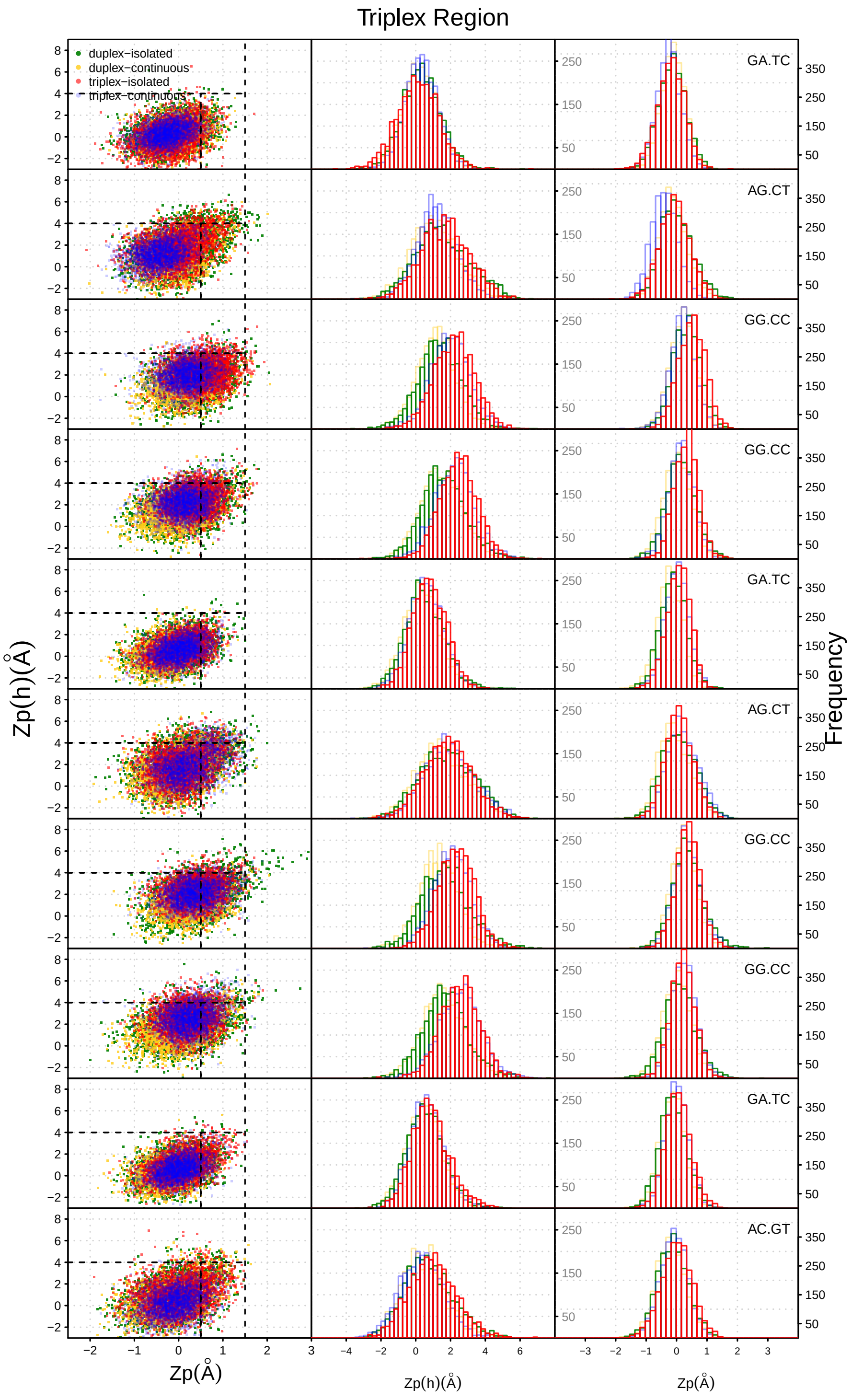

Figure S8: Inter-strand p to p vector projections on z axis of lhp reference frame and bps reference frame.

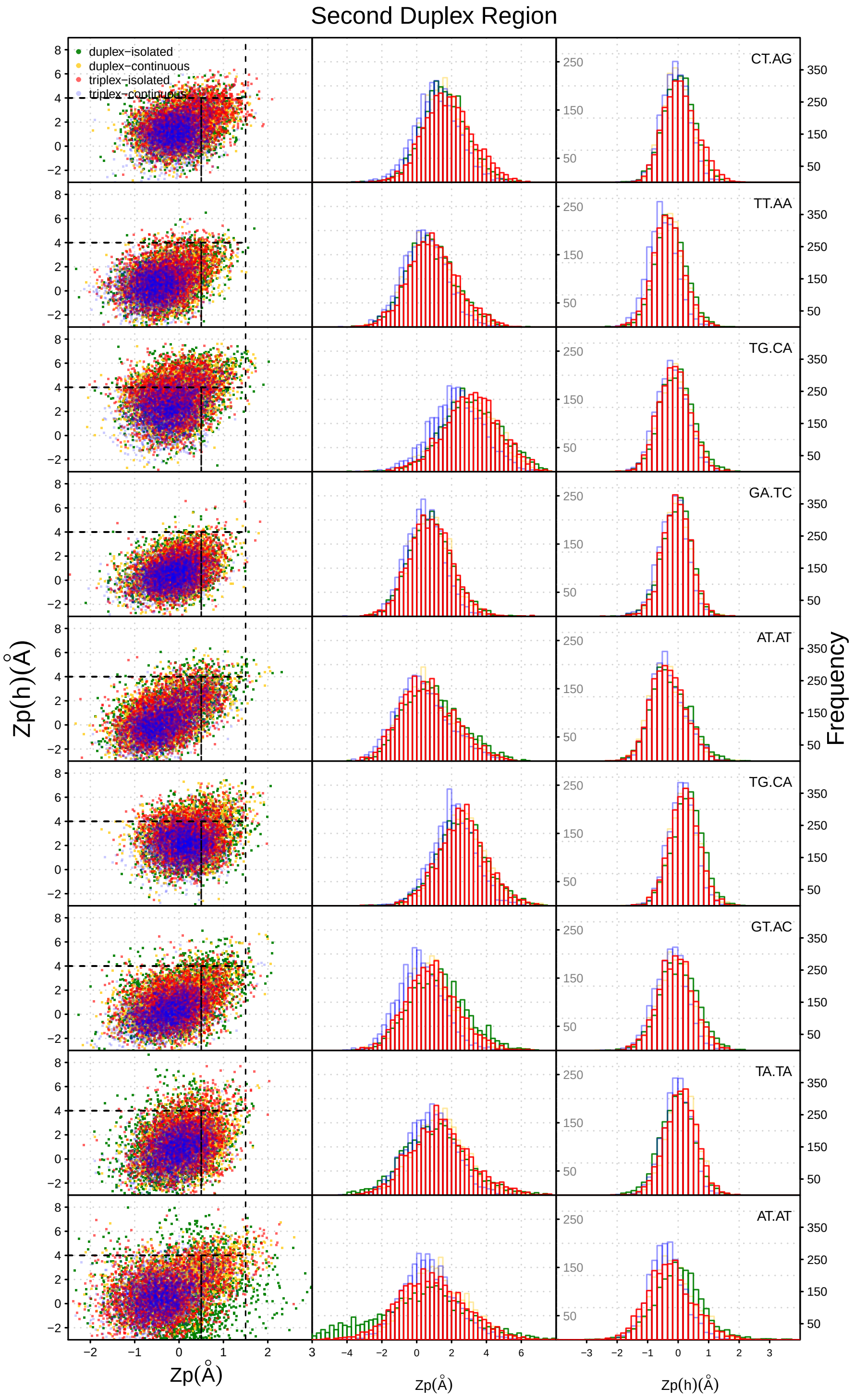

Figure S9: Inter-strand p to p vector projections on z axis of lhp reference frame and bps reference frame.

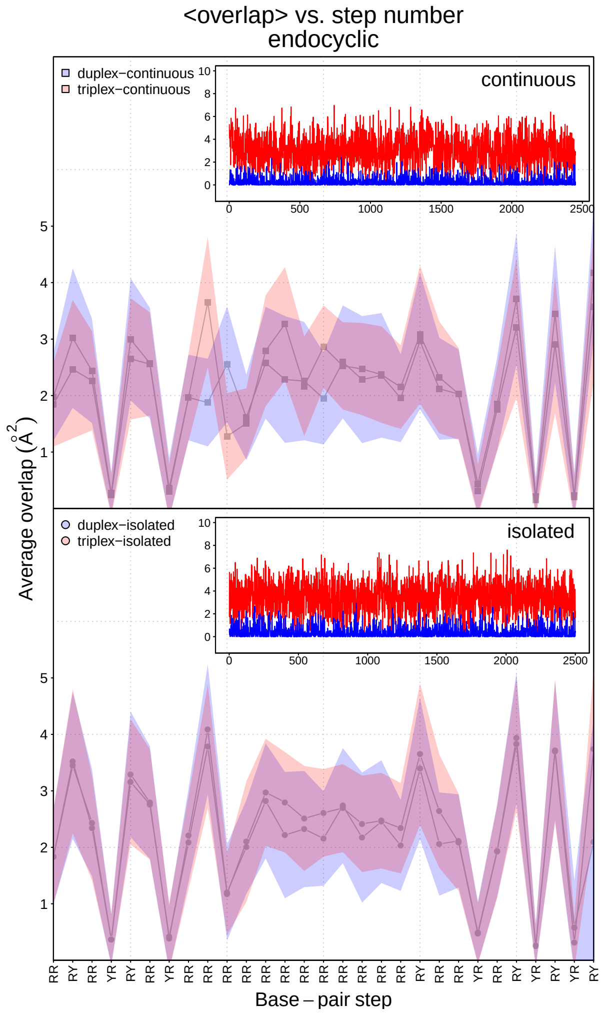

Figure S10: Average endocyclic base pair step overlap vs. base pair step number. The inset displays the overlap values along the whole trajectory for steps two (in red) and four (in blue).

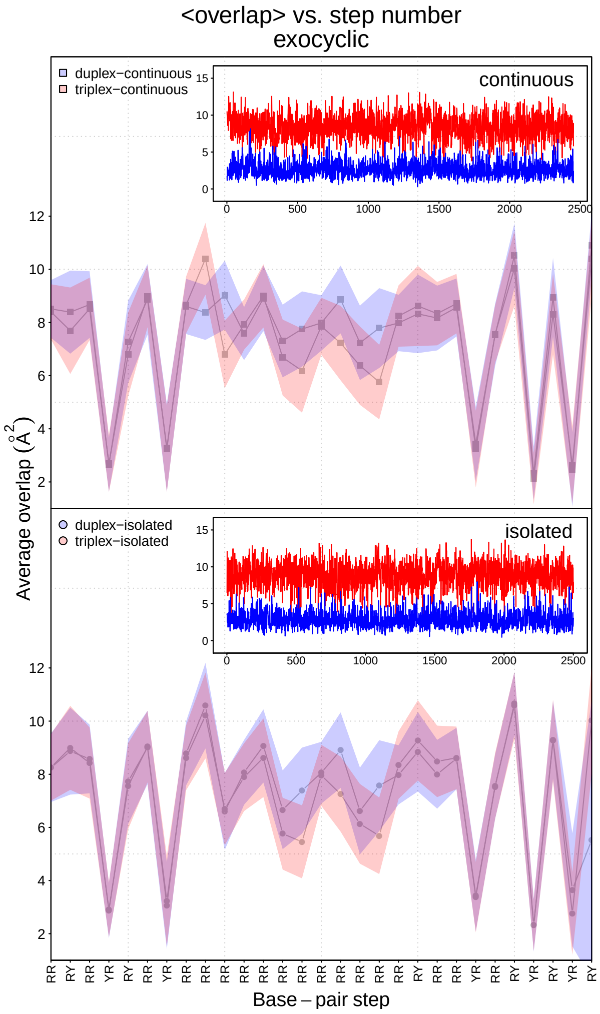

Figure S11: Average exocyclic base pair step overlap vs. base pair step number. The inset displays the overlap values along the whole trajectory for steps two (in red) and four (in blue).

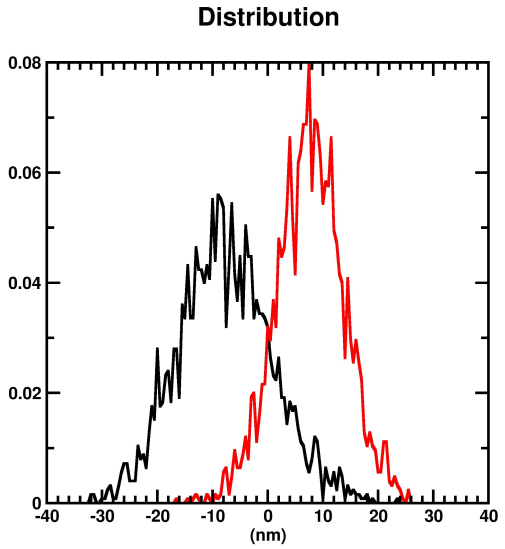

Figure S12: Distribution along the first eigenvector obtained from PCA analysis of the merge trajectory of the duplex. Values for the DNA duplex in absence and in presence of the TFO in black and in red, respectively.
